# Supplementary material for: How does a social norms-based intervention affect behaviour change? Interim findings from a cluster randomised controlled trial in Odisha, India
Source: BMJ Open. 2022 Jul 8;12(7):e053152. doi: 10.1136/bmjopen-2021-053152 (PMC9272109; doi:10.1136/bmjopen-2021-053152)
Supplement: Supplementary data [file bmjopen-2021-053152supp001.pdf]

Appendix Table 1. Regression coefficients from models predicting midline hemoglobin as a function of treatment assignment, exposure to three intervention components, and control variables

	Model 1	Model 2	Model 3	Model 4	Model 5
RANI Intervention Overall	0.12	0.14	0.11	0.10	0.07
Group education sessions					
1 (vs. 0)		0.11			0.11
2 (vs. 0)		0.19			0.17
3 (vs. 0)		0.14			0.08
4 (vs. 0)		-0.04			-0.11
5 (vs. 0)		-0.03			-0.10
6 (vs. 0)		-0.12			-0.19
7 (vs. 0)		-0.03			-0.03
8 (vs. 0)		0.08			0.06
9 (vs. 0)		-0.08			-0.09
10 (vs. 0)		0.04			0.03
11 (vs. 0)		-0.15			-0.20
Anemia testing					
1 (vs. 0)			0.11		0.12
2 (vs. 0)			-0.13		-0.14
3 (vs. 0)			-0.29		-0.31
4+ (vs. 0)			-0.19		-0.17
RANI Comm videos					
1 (vs. 0)				0.18	0.18
2 (vs. 0)				-0.09	-0.04
3 (vs. 0)				-0.02	0.08
4+ (vs. 0)				0.05	0.20
Control Variables					
Age	-0.00	-0.00	-0.00	-0.00	-0.00
Education	0.02	0.01	0.02*	0.02	0.02
Breastfeeding	-0.16**	-0.16*	-0.16**	-0.16**	-0.16**
Number of children	-0.02	-0.02	-0.02	-0.02	-0.02
Caste/Tribe	-0.39***	-0.39***	-0.39***	-0.39***	-0.39***
Knows anemia status at baseline	-0.00	-0.00	-0.00	-0.00	-0.00
Baseline IFA use	-0.39**	-0.40**	-0.40**	-0.39**	-0.40**
Non-RANI intervention exposure	0.15	0.13	0.15	0.15	0.12
R2	0.03	0.04	0.04	0.04	0.04
