# Supplementary material for: How does a social norms-based intervention affect behaviour change? Interim findings from a cluster randomised controlled trial in Odisha, India
Source: BMJ Open. 2022 Jul 8;12(7):e053152. doi: 10.1136/bmjopen-2021-053152 (PMC9272109; doi:10.1136/bmjopen-2021-053152)
Supplement: Author's manuscript [file bmjopen-2021-053152.draft_revisions.pdf]

BMJ Open is committed to open peer review. As part of this commitment we make the peer review history of every article we publish publicly available.

When an article is published we post the peer reviewers' comments and the authors' responses online. We also post the versions of the paper that were used during peer review. These are the versions that the peer review comments apply to.

The versions of the paper that follow are the versions that were submitted during the peer review process. They are not the versions of record or the final published versions. They should not be cited or distributed as the published version of this manuscript.

BMJ Open is an open access journal and the full, final, typeset and author-corrected version of record of the manuscript is available on our site with no access controls, subscription charges or pay-per-view fees (http://bmjopen.bmj.com).

If you have any questions on BMJ Open's open peer review process please email info.bmjopen@bmj.com

BMJ Open

Unpacking how a social norms-based intervention affects behavior change: The Reduction in Anemia through Normative Innovations (RANI) Project

Journal:	*BMJ Open*
Manuscript ID	bmjopen-2021-053152
Article Type:	Original research
Date Submitted by the Author:	06-May-2021
Complete List of Authors:	Sedlander, Erica<br>Pant, Ichhya<br>Bingenheimer, Jeffrey<br>Yilma, Hagere<br>Patro, Lipika<br>Mohanty, Satya<br>Ganjoo, Rohini<br>Rimal , Rajiv
Keywords:	PUBLIC HEALTH, NUTRITION & DIETETICS, Public health < INFECTIOUS DISEASES

SCHOLARONE™
Manuscripts

I, the Submitting Author has the right to grant and does grant on behalf of all authors of the Work (as defined in the below author licence), an exclusive licence and/or a non-exclusive licence for contributions from authors who are: i) UK Crown employees; ii) where BMJ has agreed a CC-BY licence shall apply, and/or iii) in accordance with the terms applicable for US Federal Government officers or employees acting as part of their official duties; on a worldwide, perpetual, irrevocable, royalty-free basis to BMJ Publishing Group Ltd ("BMJ") its licensees and where the relevant Journal is co-owned by BMJ to the co-owners of the Journal, to publish the Work in this journal and any other BMJ products and to exploit all rights, as set out in our licence.

The Submitting Author accepts and understands that any supply made under these terms is made by BMJ to the Submitting Author unless you are acting as an employee on behalf of your employer or a postgraduate student of an affiliated institution which is paying any applicable article publishing charge ("APC") for Open Access articles. Where the Submitting Author wishes to make the Work available on an Open Access basis (and intends to pay the relevant APC), the terms of reuse of such Open Access shall be governed by a Creative Commons licence – details of these licences and which Creative Commons licence will apply to this Work are set out in our licence referred to above.

Other than as permitted in any relevant BMJ Author's Self Archiving Policies, I confirm this Work has not been accepted for publication elsewhere, is not being considered for publication elsewhere and does not duplicate material already published. I confirm all authors consent to publication of this Work and authorise the granting of this licence.

For peer review only

Title: Unpacking how a social norms-based intervention affects behavior change: The Reduction in Anemia through Normative Innovations (RANI) Project

Authors: Erica Sedlander, DrPH, MPH[1], Ichhya Pant, DrPH (c), MPH[1], Jeffrey Bart Bingenheimer, PhD,[1] Hagere Yilma, PhD (c), MPH[1], Lipika Patro,[3] Satya Mohanty, MS,[2] Rohini Ganjoo, PhD[4], Rajiv N. Rimal, PhD[1, 5]

[1]The George Washington University, Milken Institute School of Public Health, Department of Prevention and Community Health
[2]DCOR Consulting
[3]IPE Global Limited
[4]The George Washington University, School of Medicine and Health Sciences, Department of Integrated Health Sciences
[5] Johns Hopkins University, Department of Health Behavior and Society

Erica Sedlander: (corresponding author)
The George Washington University, Milken Institute School of Public Health
950 New Hampshire, Department of Prevention and Community Health
Washington D.C. 20052
esedlander@gwmail.gwu.edu

Erica Sedlander: esedlander@gwmail.gwu.edu
Ichhya Pant: ipant@email.gwu.edu
Jeffrey Bart Bingenheimer: bartbing@gwu.edu
Hagere Yilma hyilma@gwmail.gwu.edu
Lipika Patro: lpatro@ipeglobal.com
Satya Mohanty: satya.dcor@gmail.com
Rohini Ganjoo: rganjoo@email.gwu.edu
Rajiv N. Rimal: rimal@jhu.edu

For peer review only

Unpacking how a social norms-based intervention affects behavior change: The Reduction in Anemia through Normative Innovations (RANI) Project

Abstract

Background: Behavior change interventions targeting social norms are burgeoning but researchers have little guidance on what they look like and which components affect behavior change. The Reduction in Anemia through Normative Innovations (RANI) project designed an intervention to increase iron folic acid consumption in Odisha, India.

Purpose: This paper examines which components affect uptake.

Methods: We collected baseline data and midline data six months later from women in the control and treatment arms (n = 3,800). Using nested models, we analyzed data from three different intervention components: monthly community-based testing for anemia, participatory group education sessions, and videos. We also examined whether age, caste, education, and communicating about the intervention moderated the effect of the intervention on iron supplement use.

Results: Residing in treatment villages increased the odds of taking supplements by more than 16 times. Being exposed to each of the intervention components separately and as reported here, all together, also increased use. Getting tested for anemia increased the odds by 38%, followed by watching videos, which increased the odds by 26%. Participating in group education sessions only increased the odds by 9%. There was no significant difference in how the intervention affected iron supplement use by age, caste, education level, or interpersonal communication about each of the intervention components.

Conclusions: All intervention components increased iron supplement use to differing degrees of magnitude. It appears that a social norms-based approach can result in improving iron folic acid uptake, though improvements in hemoglobin counts were not yet discernible.

Keywords: social norms, behavior change, intervention, anemia

Strengths and limitations of this study

- The intervention is based on formative research, theory, and co-designed with input from community stakeholders
- Data come from a large double blinded cluster-randomized controlled trial
- Intervention components are qualitatively described and then quantitatively evaluated to decipher their individual effect on iron supplement use
- We rely on self-report for iron supplement use and image recall for participation in intervention activities

Introduction

Social norms, defined as informal rules of behavior considered acceptable in a group or society [1], are increasingly recognized as drivers of or barriers to behavior change. In a review of social norms-based interventions over the last three decades, more than half were published in the last decade [2]. Recent social norms interventions in low to middle income countries have focused on changing harmful behaviors, such as intimate partner violence [3-4], female genital cutting [5], and child marriage [6]. Past research shows that people are more likely to engage in a behavior when they believe many others also do so, and when there is a social expectation that they themselves should comply. In the anemia prevention context recent literature indicates a seemingly simple behavior, e.g., taking a weekly iron supplement, is embedded in social and cultural dynamics that dictate which health behaviors are appropriate [7-8].

The theory of normative social behavior (TNSB) highlights the critical role that social norms can play in influencing health behaviors and the circumstances under which they may do

For peer review only - http://bmjopen.bmj.com/site/about/guidelines.xhtml

so [9-10]. According to the TNSB, the effects on behavior of descriptive norms (i.e., one's perceptions about others' behaviors) and injunctive norms (i.e., one's perceptions of social expectations regarding the behavior) may depend upon other factors at the individual, behavioral, and environmental level [11]. Factors that could either augment or attenuate the effect of social norms messaging on iron folic acid (IFA) use include perceptions that one is at risk of getting anemia, outcome expectations that taking IFA will result in health benefits like preventing anemia or reducing fatigue, and access and availability of IFA. Therefore, interventions must aim to improve both social norms around a behavior along with potential moderators that may strengthen the relationship between social norms and behaviors. In a 2018 meta-analysis of social norms approaches to behavior change, Dempsey and authors found that the most effective social norms manipulations take place in one's own environment (e.g., a field trial), those that deliver messages in multiple formats, and those that target collectivist groups [12].

Many social norms-based interventions are often complex and resource intensive. To implement them well, program planners need to understand the social context, the social norms around the behavior of interest, and barriers and facilitators at multiple levels of the socio-ecological model [13,14]. Guided by the TNSB, the Reduction in Anemia through Normative Innovations (RANI) Project conducted formative research to delineate significant facilitators to and barriers of iron folic acid consumption in Odisha, India [8]. Findings from that work led to the design of the intervention, which included three components: (a) hands-on participatory learning modules conducted in small groups, (b) dissemination of short videos focusing on iron consumption norms, and (c) monthly hemoglobin testing for anemia, followed by public display of (anonymized) community results.

For peer review only - http://bmjopen.bmj.com/site/about/guidelines.xhtml

In this paper, we seek to determine which intervention components impact iron folic acid consumption at midline. This knowledge allows us to adapt the intervention implementation according to empirical findings. Because social and political realities on the ground cannot be fully predicted at the outset of any given field trial [15], we deemed this approach more preferable than simply implementing a static intervention based on *a priori* data. Thus, the primary goal of this paper is to examine the extent to which each intervention component contributed to the overall effects of the intervention. In addition, because the influence of the intervention may be different across subgroups, we also examine how susceptibility to intervention impact varied by age, caste, education, and communication activity. We hope that delineating the effects of each intervention component will provide guidance for future social norms-based intervention designs. As Davis et al. ([69], p. 2218) argue, "while the social norms approach is based in a rich theory, the theory does little to illuminate implementation details of interventions [16]."

Methods

This study was approved by the George Washington University Institutional Review Board (FWA00005945), Sigma Science and Research, an independent IRB located in New Delhi, India, and the Indian Council for Medical Research's Health Ministry's Screening Committee.

Patient and Public Involvement Statement

Key stakeholders from the community where the intervention was implemented participated in a two-day convening in Bhubaneswar, Odisha to co-design the intervention. Community health workers helped to implement the intervention and disseminate findings back

For peer review only - http://bmjopen.bmj.com/site/about/guidelines.xhtml

to the community. Patients were not involved in the study – participants were living in the villages where the intervention took place.

Study Setting

Nestled in the eastern coast of India, the state of Odisha is predominantly rural. Most residents (95%) are Hindu, with 23% belonging to specific tribes and practicing tribal culture (NFHS 2015-2016). At approximately 2.1 children per woman, the total fertility rate sits fairly low. Our focal district, Angul, one of 30 in the state of Odisha, has almost 2,000 villages with a total population of just over 1.2 million (Government of Odisha, 2019). Men's literacy rate (87%) is higher than that of women's (70%). Almost a quarter of girls (22%) marry before age 18 and around half of married women of reproductive age use modern methods of family planning [17].

Intervention Development

To develop the RANI intervention, we conducted formative research that examined social norms around IFA use. Between March and May 2018, we collected data from four villages in the two adjacent blocks (administrative units below the district) where the intervention took place (Kishorenagar and Athamalik). We conducted 16 focus groups and 21 individual interviews (n = 148), stratified by age and gender, with women of reproductive age, husbands, mothers-in-law, and key informants. To explore women's social norms within the focus groups, we used [18]. Vignettes can also help uncover if social sanctions exist and unpack existing social norms. Four researchers, two from India and two from the United States, analyzed transcripts using NVivo v.12 to identify barriers and facilitators to IFA use.

For peer review only - http://bmjopen.bmj.com/site/about/guidelines.xhtml

We found that social norms and available services varied substantially for pregnant women, non-pregnant women, and adolescents. Specifically, we found that most participants believed only pregnant women and adolescents in school consume IFA (descriptive norms). Participants also stated that only pregnant women and those diagnosed with anemia should be taking IFA regularly (injunctive norms). Furthermore, we found that frontline health workers only distributed IFA to pregnant women. Adolescents enrolled in school can also obtain them weekly [8].

Non-pregnant women (our sample for this paper) were not receiving IFA from frontline health workers. Indeed, barriers faced by non-pregnant women were significant: they needed to visit a health center, get tested for anemia, and then obtain the IFA if they were diagnosed as anemic. We also found that risk perception was low, with most participants believing that only "a handful of women" in their community had anemia when, in reality, more than half of women are anemic. When anemia was referenced, we found that participants were primarily referring to severe anemia, not its mild or moderate forms [8].

Our findings also revealed that inequitable gender norms were an upstream barrier to women's accessing and adhering to IFA supplements. Specifically, women prioritized their family's health and well-being over their own, normalized fatigue as part of a woman's plight, and given that they often do all of the household works and also work outside of the home, they lacked time (and often autonomy) to visit a health center on their own [19-20].

We used findings from the qualitative research, past literature on anemia reduction efforts, and the TNSB to design the RANI intervention. We also held a three-day convening in Bhubaneswar, Odisha, where we invited frontline health workers from the community, anemia researchers, and program planners to co-design an effective social norms-based intervention. The

For peer review only - http://bmjopen.bmj.com/site/about/guidelines.xhtml

RANI Project intervention comprises three main components, each tapping into social norms differently: participatory learning modules; RANI Comms (videos), and community hemoglobin testing. A description of all RANI activities can be found in **Table 1**.

Study Design

Data for this study come from the RANI Project [21]. The RANI project uses a cluster randomized controlled trial to evaluate the efficacy of a norms-based intervention to increase IFA use and reduce anemia in Odisha, India. The RANI project selected two blocks within the Angul district in the state of Odisha. We grouped contiguous villages in these blocks into clusters, which we then randomly assigned to either the treatment or control arm. Villages in the treatment arm were exposed to the RANI intervention, while villages in the control arm continued with “care-as-usual”. We created clusters to minimize contamination; clusters were separated by either a natural buffer (i.e., mountain or river) or a village that was neither in the treatment nor in the control arm. This process resulted in a total of 89 clusters from 239 villages. We then segmented clusters by the proportion of caste/ethnic groups (in India they are called scheduled castes and scheduled tribes) and then selected 3 per stratum, for a total of 15 clusters per arm to be included in data analysis (which comprised 81 villages).

In this paper, we report results from the baseline and midterm assessment, which is a longitudinal study from both the treatment and control clusters. The response rate for the midline questionnaire was 96.2%. Interviewers visited homes up to three times and the primary reason for not taking the midline survey was not being home when the interviewers visited their house.

Participants

In each designated village, we first enumerated all households and then randomly selected households for data collection using proportion-to-size principles based on cluster

For peer review only

population. From the selected home, one woman of reproductive age (between 15 and 49 years old) was chosen (randomly if more than one woman was eligible in the same home). Although our sample consisted of 3,953 participants, this paper restricts the sample to those who were not pregnant at the time of the midline survey (n = 3,800). We do so because the primary dependent variable, taking iron and folic acid tablets, has been heavily promoted among pregnant women by the Government of India. Pregnant women are also enrolled in the health system, where physicians or community health workers provide free IFA. This is not the case among non-pregnant women, who have not been targeted as IFA recipients on the ground despite WHO and Indian government recommendations [22-23]. The demographic profile of participants included in our analysis is shown in Table 2.

Procedure

Local data collectors obtained informed consent from all individual participants included in the study in the local language, Odiya. Participants under the age of 18 were required to obtain the written permission of one parent or legal guardian. Data collectors orally administered a one-on-one survey to all participants, which assessed demographic information, psychosocial factors, and anemia-related behaviors.

Inclusion Criteria

Women were eligible for inclusion in the study if they were between the ages of 15 and 49, spoke Odiya, lived in the data collection villages (either treatment or control), and did not plan to move in the next year (as this is a longitudinal study).

Measures

Dependent Variables. Our study has two dependent variables: self-reported IFA use and objectively measured serum hemoglobin levels. We measured *IFA use at midline* using the

For peer review only

interview question, "Have you ever eaten/taken an iron tablet or syrup." (The interviewer then held up the packet of IFA tablets for the interviewee to see). We coded this as a dichotomous variable, scored 1 if currently taking and 0 if not or did in the past but stopped now. We obtained *hemoglobin levels* from all participants at midline through point-of-care hemoglobin tests using a HemoCue photometer (in line with India's National Family Health Survey methodology). This instrument provides hemoglobin levels immediately and accurately [24].

Independent Variables. We examined four independent variables. The first is a dichotomous indicator of *treatment assignment* that takes the value 1 for participants residing in intervention villages and 0 for those residing in control villages. The other three independent variables are participants' self-reports of exposure to different components of the RANI intervention. To measure exposure to the *participatory learning modules*, we took the sum of responses to six questions about how often participants had seen materials from these sessions. We used visual images from the sessions, with higher scores indicating more exposure or more frequent exposure (not seen = 0, seen once or twice = 1, and seen more than twice = 2). One question asked whether or not they had participated in any of the games that were also a part of the RANI participatory group sessions (scored as No=0 and Yest=1). Less than 1% of participants (*n=28*) marked "don't know" (which was coded as missing). We assessed participants' *own anemia testing* with the question, "How many times have you been tested for anemia as part of the RANI intervention in the last six months?" Response options ranged from 0 (never) to 4 (more than 3 times). We measured exposure to the *RANI comm videos* as the sum of responses to four questions about which of the four videos they had watched. Interviewers shared an image from each video and a brief description of the story plot. Responses were treated as dichotomous for each video (No = 0, Yes = 1) and summed across the four videos for a range from 0 to 4. We

For peer review only - http://bmjopen.bmj.com/site/about/guidelines.xhtml

assessed *communicating with others about intervention components* with three separate questions: “Talked about blood tests when talking about RANI to friends or family;” “Talked about videos when talking about RANI to friends or family;” and “Talked about RANI meetings (group education modules) when talking about RANI to friends or family.” For each, an affirmative response was coded 1 and a negative response was coded 0.

Control Variables. We asked respondents their *age*, highest completed level of *education*, and whether they belonged to a *scheduled tribe*. *IFA use at baseline* was assessed exactly as described above for midline. We asked respondents about the *number of children* they had and whether or not they were *breastfeeding*. Additionally, to understand if participants had been exposed to another intervention that was not affiliated with RANI (to avoid contamination), we asked participants, “Did you hear anything about nutrition or iron tablets from the Swabhimaan or any other program?” We coded this as a dichotomous variable (No or don’t know = 0 and Yes = 1).

Statistical Analysis

We conducted our analyses in four steps. First, we calculated frequencies and descriptive statistics of all key analytic variables by treatment and control arm, and obtained p-values testing the null hypothesis of no difference between the two arms via independent samples t-tests and Pearson’s chi-squared tests of independence for continuous and categorical variables, respectively. Second, we ran linear regressions to examine if exposure to each intervention component differed by sub groups, including older vs. younger (above 32 years old versus below 32 years old), belonging to a scheduled caste or not, and more or less educated (completed primary school or not). Third, we ran logistic regression models to examine how each intervention component individually and additively affected IFA use at midline. We show nested

For peer review only - http://bmjopen.bmj.com/site/about/guidelines.xhtml

models to demonstrate how each intervention component (including being in the treatment arms versus control arm) affected IFA use differently. All models included control variables: age, education, currently breastfeeding, number of children, whether or not they belong to scheduled caste and tribe, knowing anemia status at baseline, IFA use at baseline, and whether or not the participant reported exposure to a non-RANI intervention. The first model included being a part of the RANI intervention (treatment versus control), which we kept in all subsequent models, after which we added exposure to each individual intervention component (models 2-4), and then all of the intervention components together (model 5). Finally, we ran logistic regression models that included interaction terms between communicating about the intervention and age, education, and caste. We used STATA, version 14 to conduct all analyses. To obtain a robust variance estimate that adjusts for within-cluster correlation, we used the Huber-White clustered standard errors command [25].

Results

Description of the sample included in our study is shown in Panel A of Table 2. Average age was 31 years old and between a quarter and a third of participants were a part of the tribal population. On average, participants completed primary school (6 years of education). Participants in both treatment and control arms had on average more than one child and fewer than two. About 20% of women in both arms were currently breastfeeding. The only statistically significant difference between the treatment and control arms was age. Women in the treatment arm were 31 years old versus 30 years old in the control arm ($p < .05$). All other demographics were not statistically different by treatment and control. Panel B of Table 2 shows that exposure to non-RANI interventions was low in both study arms.

For peer review only - http://bmjopen.bmj.com/site/about/guidelines.xhtml

Crude estimates of the effects of the RANI intervention at midline are shown in Panel C of Table 2. Participants from the treatment arm reported more IFA use at midline (31.6%) compared to the control arm (3%) (p = < .001). However, hemoglobin levels were not statistically different in the two arms (11.7% versus 11.5%) (p =.14). Panel D of Table 2 shows that exposure to each of the intervention components was widespread among participants in the treatment arm and low among participants in the control arm. Specifically, 88% of participants in the treatment arm compared to only 13% in the control arm reported that they attended at least one group educational session (p = <.001). Eighty percent of participants in the treatment arm reported that they had been tested at least once as part of the intervention compared to 0% in the control arm (p = <.001). Similarly, 80% of women in the treatment arm reported that they watched one or more RANI comm videos, compared to less than 1% in the control arm (p = <.001). Lastly, exposure to interventions other than RANI was minimal across both the treatment and control arms (1.2% and 2.1%, respectively).

Table 3 shows that older women attended more educational sessions (2.58 compared to 2.28; p = < .001), and had more exposure to educational videos than younger women (1.33 compared to 1.15; p = < .001). Hemoglobin testing did not vary by age. Table 3 also shows that neither educational attainment nor caste or tribal background was associated with exposure to the intervention.

Because there was no crude difference between the treatment and control arms in hemoglobin level at midline (see Panel C of Table 2), we did not conduct analyses linking exposure to specific intervention components to hemoglobin levels. However, nested logistic regression models predicting self-reported IFA use as a function of study arm and exposure to each of the intervention components are presented in Table 4. As seen in Model 1, after

For peer review only - http://bmjopen.bmj.com/site/about/guidelines.xhtml

controlling for demographic variables and IFA use at baseline, simply being in the treatment arm increased a woman's odds of taking IFA by more than 16 times (OR = 16.73; p = < .001). In Model 2, we added exposure to the group education sessions and found that each session that a woman attended increased her odds of taking IFA by 23 percent (OR = 1.23; p = < .001). Model 3 suggest that undergoing an additional test for anemia is associated with a 75 percent increase in the odds of taking IFA (OR = 1.75; p = <.001). Model 4 similarly shows that for each RANI Comm video that a woman watched (1-4 total), her odds of taking IFA increased by almost 50% (OR 1.49; p = < .001). Model 5 with all intervention components included shows that while all effect sizes drop, each component still had a significant effect on IFA use including: attending group education sessions (OR 1.09; p < .01), getting tested for anemia (OR 1.38; p < .001); and watching RANI comm videos (OR 1.26; p < .001). This indicates that each component explains a unique part of the variance in current use of IFA. Our final logistic regression model is not shown in Table 3 but included interaction terms between each of the three intervention components and age, education, and caste. None of the interaction terms were statistically significant.

Discussion

In this paper we present findings from the midline assessment of a multicomponent behavioral intervention intended to increase IFA use and decrease anemia among women of reproductive age in Odisha, India. We find strong evidence that the intervention increased the prevalence of self-reported IFA use among non-pregnant women, but no evidence of an effect of the intervention on average hemoglobin levels in that group. Self-reported exposure to all three intervention components was high among women in the treatment arm, which did not vary substantially by education or membership in a scheduled caste or tribe. However, exposure to

For peer review only - http://bmjopen.bmj.com/site/about/guidelines.xhtml

group participatory sessions and videos was higher among older women than among younger women. Self-reported exposure to all three intervention components was positively associated with IFA use, and these exposures accounted for most but not all of the overall effect of the RANI intervention on IFA use among non-pregnant women.

Our success in impacting older women could be due to a number of reasons. In the Angul district, older women hold more power in their households and control over their own life choices than younger women. In addition, older women have more disposable time in their day-to-day lives, compared to younger women, to be able to attend our time-intensive sessions. Our own observations in the villages lead us to believe that daughters-in-law devote more time in completing household chores, compared to mothers-in-law who, by their higher status in the family hierarchy, have the autonomy to leave home (to attend RANI sessions, for example). Additionally, their children may be grown and thus they have more time to attend RANI intervention activities and to focus on their own health and well-being. Newly married women often face social restrictions to participate in public meetings.

This finding has an important implication for interventions in rural India. One (albeit untested) strategy is to think of older women as direct audience members for interventions, who are then trained to convey intervention messages to their younger counterparts at home. Because they tend to occupy a higher status in the social hierarchy, they may also have higher credibility and thus be able to “sell” the intervention messages more effectively (than, say, younger women in the community). Thus, older women can serve as ambassadors of the intervention. To our knowledge, this has not been an explicit intervention strategy in rural India, but our findings point to the possibility that it could be an effective one.

We also found that neither education nor tribal membership was associated with exposure to the intervention. Put another way, it appears that the RANI Project's outreach efforts did not disproportionately favor those with higher privilege in society. Indeed, even though exposure to the intervention did not differ by tribal status or education, in our multivariate models we found some evidence that those will lesser education had higher IFA uptake. We view this finding rather optimistically, given that interventions have the potential for exacerbating existing differences in society along access and socioeconomic lines [26], as has been documented by the literature on the knowledge gap hypothesis [27-28].

We saw strong evidence that testing women for anemia (through finger-prick hemoglobin counts) was associated with IFA uptake: those who got tested more often were more likely to consume IFA tablets. The reasons for this are not known precisely, but we do have a few explanations. First, it could be that testing revealed to women the low hemoglobin counts, which spurred them to act to improve their scores. Our formative assessment had revealed that feeling weak was often a part of women's self-identity, that women often believed that fatigue was par for the course [19] and that this was part of the gendered norms for women [20].We suspect that, in contrast, an objective measure of iron in the blood, observed through hemoglobin testing, quantified and thus provided precision to a phenomenon woman had come to accept as a vague notion of fatigue. We found that testing was highly demanded and motivational. Indeed, our process evaluation data indicate that, despite a high (60%) coverage rate of anemia testing for women in our intervention catchment areas, we have been unable to meet demand for testing and repeat testing.

A second reason why testing may have motivated women to consume IFA tablets pertains to observational learning around social norms. Because test results were displayed

publicly in the community, and most educational sessions linked the test results with IFA consumption, women likely came to link their consumption behaviors with hemoglobin readings – not only for themselves but also for what they had observed among others in their community, where hemoglobin testing was a monthly event. These events were so popular that, in some villages, the waiting list for testing ran up to 90 names (we were unable to accommodate more than 15 women per village per month, given the study design and resources). This public display of IFA testing and hemoglobin results also normalized women prioritizing their own health potentially shifting gender norms that solely focus on pregnant women's health or the health of the family. Although these underlying reasons are somewhat speculative, they do point to the need to study more precisely the link between testing and IFA consumption.

We also found that exposure to the communication videos was significantly associated with increased IFA uptake. Indeed, each additional video exposure was associated with a 26% higher likelihood of taking IFA tablets. We suspect that the underlying reason for this finding pertains to another normative component of the intervention. The four videos we developed targeted different audiences, including adolescents, husbands, mothers-in-law, and women of reproductive age. As an explicit campaign strategy, each video was shown to each of the parties, including those who were not the explicit target audience for the particular video. This was done so that each group came to understand that the other groups were also being targeted by the intervention. So, for example, adolescent girls saw that there were videos that also addressed men and older women. Similarly, men saw that the videos targeted other men and other women. The overall strategy was to communicate the message that the entire community was engaged in the task of reducing anemia.

Finally, we should note that attending each educational session, though statistically significant, only increased the likelihood of IFA consumption by nine percent. Although this magnitude seems small, we still consider it as important. These sessions were designed primarily to raise awareness and improve knowledge about anemia, iron-rich foods, and diet diversity. We also included discussions about gender roles, how eating order in the home disfavors women's health, and the need to remain strong to be able to take care of others. We have few reasons to believe that knowledge in these domains would directly translate into behaviors. However, a significant body of work demonstrates that knowledge about health is a necessary, though not sufficient, condition for behavior change [29 - 30] and that people often hold knowledge in abeyance, to be acted upon at the appropriate time [31]. From this perspective, it seems that educational sessions may have played an important role, even if their impact on behavior change was small.

Of course, our study is not without limitations. First and foremost is the apparent lack of an effect of the RANI intervention on hemoglobin use, combined with the self-reported nature of our assessment of IFA use. One plausible explanation of these disparate findings is that the increase in IFA use among members of the treatment arm is real, but is not sufficient in magnitude or duration to produce a corresponding increase in hemoglobin levels. Alternatively, it could be attributable to differential misreporting of IFA use among treatment group members, a form of courtesy or social desirability bias. Findings from the end line data collection, planned to take place at the end of the project, may help to adjudicate between these two explanations. A related limitation is that, while treatment assignment is objectively measured on the basis of project administrative data, exposure to the three intervention components is self-reported and may be subject to some level of misreporting. Additionally, although our study used a

For peer review only - http://bmjopen.bmj.com/site/about/guidelines.xhtml

representative sample of areas and women in our focal areas, findings may not be generalizable outside of that setting.

In spite of these limitations, our study has strengths: the intervention is based on formative research; it is co-designed by stakeholders from the area; we use a theoretically driven and adaptive approach; and the study design itself is robust, with an underlying randomization to experimental arms.

Conclusion

Our findings show that a social norms-based intervention can be successful in increasing IFA use. They also demonstrate that unique intervention components separately and altogether impact this success. All three components appear to contribute something to the overall effect of the intervention. While all three intervention components tapped into social norms messaging, hemoglobin testing provided individual health information, village level health information, a comparison to other villages, and changes in health information over time. This multi-level component, coupled with the other two components, may help women reach the tipping point to take and adhere to IFA. While IFA use shows promise, hemoglobin levels may need more time to show significant changes, especially among non-pregnant women who were not taking IFA and who only take it weekly. End line results will elucidate more information about the full RANI intervention effects.

For peer review only - http://bmjopen.bmj.com/site/about/guidelines.xhtml

Trial Registration
This trial was registered with Clinical Trial Registry- India (CTRI) (CTRI/2018/10/016186) on 29 October 2018.

Funding
This work was supported by a grant from The Bill and Melinda Gates Foundation (OPP1182519) to the George Washington University, Rajiv N. Rimal, principal investigator. The funders had no role in study design, data collection and analysis, decision to publish, or preparation of the manuscript

Author Contribution Statement
ES: Conceptualization, methodology, analysis, writing – original draft
IP: Conceptualization, methodology, writing – review & editing
JB: Methodology, writing – review & editing
HY: Methodology, data curation, writing – review & editing
LP: Project administration, supervision, writing – review & editing
SM: Project administration, supervision, writing – review & editing
RG: writing – review & editing
RR: Conceptualization, methodology, supervision, funding acquisition, writing – review & editing

Data sharing statement:

Data used for this study can be found in our online data repository: GW Scholarspace (https://scholarspace.library.gwu.edu/) and can also be requested from the first author.

Table 1. Description of RANI intervention activities

**Group participatory learning sessions**<br><br>We developed ten one-hour-long group participatory learning modules on various topics related to anemia prevention, including iron folic acid supplementation, diet diversity, and social norms/gender norms that may impact a woman's ability to take iron supplements. These monthly group participatory learning sessions are delivered through in-person activities and games. Women and their social networks (e.g., husbands and mothers-in-law) were all invited to participate so that women and those important to her are being exposed to the same messaging.
**RANI Comm videos**

For peer review only - http://bmjopen.bmj.com/site/about/guidelines.xhtml

We created RANI Comm videos in the local language, Odiya, with local residents as actors. These videos highlighted the stories of women overcoming barriers related to IFA consumption and other social norms prevalent in the area. The videos were shown on smartphones and tablets to both individuals and small groups. The videos targeted various audiences (pregnant women, non-pregnant women, husbands, and mothers-in-law) and addressed barriers and facilitators to IFA use that we identified in the formative research followed by group discussion sessions. Descriptive and injunctive norms messages around IFA consumption were included in each storyline. An example of injunctive norms messaging in the storyline is a mother-in-law expressing that her daughter-in-law should be taking care of herself too, not just looking after the family, and should be taking weekly IFA to avoid anemia even if she's not pregnant.
**Community based hemoglobin testing**<br>We also conducted monthly anemia testing of the women using a digital Hemocue meter. These instant results are shared at the individual, group, and village levels with the help of blood shaped cards (different colors indicating anemia severity) and infographics appropriate for a low literacy population. Monthly community-based testing was followed by a discussion about trends in anemia and village-level comparisons (based on the hemoglobin readings) with neighboring communities at both the individual and community levels. This provides ipsative feedback (information people receive about their ongoing progress over time), normative feedback (information about the particular individual's achievements relative to those of her social peer), and aspirational feedback (comparisons people make between their current state of affairs and the goals they may have set for themselves).

For peer review only - http://bmjopen.bmj.com/site/about/guidelines.xhtml

Table 2. Description of Participants in Treatment and Control Arms

	**Treatment (n= 1,903)**	**Control (n = 1,897)**	**P >|t|**
A. Demographic Background			
Age (years)	31.2	30.5	**0.02**
Part of the tribal population	23.4%	31.2%	0.14
Education (years)	6.09	6.17	0.78
Currently breastfeeding	21.3%	21.8%	0.74
Number of children	1.69	1.69	0.97
B. Exposure to a non-RANI related intervention	1.2%	2.1%	0.16
C. Dependent Variables			
IFA use at midline (yes/no)	31.6%	3.0%	**0.00**
Hemoglobin at midline (g/dl)	11.66	11.51	0.14
D. Exposure to Intervention Components			
Attended at least one group education session	88%	13%	**0.00**
Hemoglobin testing (tested at least once)	80%	0%	**0.00**
Watched at least one RANI Comm video	80%	0.2%	**0.00**

Notes: *T*-tests compare demographic differences between treatment versus control arms. Significant cells are in bold. IFA = iron folic acid. *T*-tests compare IFA use and exposure to different intervention components by treatment and control arms. Significant cells are in bold. The other non-RANI related intervention includes anemia related education as one component that may have had some overlap with our intervention. We included this as a control in our models to ensure that any changes in behavior are a result of our intervention alone.

For peer review only

Table 3. Exposure to each intervention component by age, education, and caste (treatment arm only)

	Average number of group educational sessions attended	Proportion undergoing at least one hemoglobin test	Average number of RANI Comm videos seen
Age			
Less than 32 years	2.28 (SD: 2.92)	0.63 (SD: 0.97)	1.15 (SD: 1.61)
32 years or more	2.58 (SD: 3.16)	0.63 (SD: 0.91)	1.33 (SD: 1.69)
p-value	**0.00**	0.92	**0.00**
Education			
Up to completed primary	2.41 (SD: 3.10)	0.59 (SD: 0.89)	1.25 (SD: 1.67)
Greater than completed primary	2.42 (SD: 2.97)	0.66 (SD: 0.99)	1.21 (SD: 1.62)
p-value	0.96	0.14	0.64
Scheduled Tribe or Caste			
Yes	2.15 (SD: 3.00)	0.55 (SD: 0.91)	1.08 (SD: 1.60)
No	2.50 (SD: 3.03)	0.66 (SD: 0.96)	1.28 (SD: 1.66)
p-value	0.29	0.22	0.27

*SD = Standard deviations

For peer review only

Table 4. Adjusted Odds Ratios from Logistic Regression Models Predicting Iron Folic Acid (IFA) Use at Midline from both Treatment and Control arms Combined

	Model 1	Model 2	Model 3	Model 4	Model 5
RANI Intervention overall	16.73***	6.60***	7.99***	5.91***	4.07***
Intervention Components					
Group educational sessions		1.23***			1.09**
Anemia Testing (Hb)			1.75***		1.38***
RANI Comm videos				1.49***	1.26***
Control Variables					
Age	0.99	0.99	1.00	0.99	1.00
Education	0.97	0.97	0.96*	0.98	0.97*
Breastfeeding	1.36*	1.37*	1.41**	1.41**	1.42**
Number of children	1.00	0.96	0.96	0.98	0.97
Caste/Tribe	1.31	1.28	1.30	1.30	1.28*
Knows anemia status at baseline	1.00	1.00	1.00	1.00	1.00
Baseline IFA use	2.48***	2.86***	2.60***	2.72***	3.03***
Non-RANI intervention exposure	1.87	1.00	1.71	1.60	1.45
R^2	0.19	0.23	0.23	0.23	0.25

Notes: *$p < .05$, **$p < .01$, ***$p < .00$

For peer review only

References

1. Reno, R. R., Cialdini, R. B., & Kallgren, C. A. (1993). The trans situational influence of social norms. *Journal of personality and social psychology*, *64*(1), 104.
2. Yemin, P., Fei, M., Lahlou, S., & Levy, S. (2019). Using social norms to change behavior and increase sustainability in the real world: A systematic review of the literature. Sustainability, 11(20), 5847.
3. Simone Francis, Prabin Nanicha Shrestha, Binita Shrestha, Gemma Ferguson , Brian Batayeh, Monique Hennink & Cari Jo Clark (2020): The Influence of Organised Diffusion on Social Norms Change: Addressing Intimate Partner Violence in Nepal, Global Public Health, DOI: 10.1080/17441692.2020.1845767
4. Clark, C. J., Bergenfeld, I., Batayeh, B., Ferguson, G., Shrestha, B., & Shrestha, P. N. (2019). Intimate partner violence in Nepal: Change starts at home study, what works to prevent violence: A global programme to prevent violence against women and girls evidence brief. South Africa Medical Research Council.
5. Shell-Duncan, B., Wander, K., Hernlund, Y., & Moreau, A. (2011). Dynamics of change in the practice of female genital cutting in Senegambia: testing predictions of social convention theory. Social science & medicine (1982), 73(8), 1275–1283. https://doi.org/10.1016/j.socscimed.2011.07.022
6. Vaitla, Bapu, Alice Taylor, Julia Van Horn, and Ben Cislaghi. 2017. Social Norms and Girls' Well-Being: Linking Theory and Practice. Washington, D.C.: Data2X.
7. Nagata, J. M., Gatti, L. R., & Barg, F. K. (2012). Social determinants of iron supplementation among women of reproductive age: a systematic review of qualitative data. Maternal & child nutrition, 8(1), 1-18.
8. Anonymous 2020
9. Rimal, R. N., Lapinski, M. K., Cook, R. J., & Real, K. (2005). Moving toward a theory of normative influences: How perceived benefits and similarity moderate the impact of descriptive norms on behaviors. Journal of health communication, 10(5), 433-450.
10. Rimal, R. N., & Real, K. (2005). How behaviors are influenced by perceived norms: A test of the theory of normative social behavior. Communication research, 32(3), 389-414.
11. Chung, A., and Rimal, R. N. (2016). Social norms: a review. *Rev. Commun. Res.* 4, 1–29. doi: 10.12840/issn.2255-4165.2016.04.01.008
12. Dempsey, R., McAlaney, J., Bewick, B. (2018). A Critical Appraisal of the Social Norms Approach as an Interventional Strategy for Health-Related Behavior and Attitude Change. Frontiers in Psychology, 6. https://doi.org/10.3389/fpsyg.2018.02180
13. Cislaghi, B., Nkwi, P., Mackie, G & Shakya, H. (2020). Why context matters for social norms interventions: The case of child marriage in Cameroon, Global Public Health, 15:4, 532-543, DOI: 10.1080/17441692.2019.1704818
14. Edberg, M., & Krieger, L. (2020). Recontextualizing the social norms construct as applied to health promotion.. *SSM-Population Health, 10* (). http://dx.doi.org/10.1016/j.ssmph.2020.100560
15. Vishwanath K., Synowiec C & Agha S. Responsive feedback: Towards a new paradigm to enhance intervention effectiveness. (2019). Gates Open Research, 3:781.
16. Davis, C.A.; Heiman, J.R.; Menczer, F. A Role for Network Science in Social Norms Intervention. Procedia Comput. Sci. 2015, 51, 2217–2226.

For peer review only - http://bmjopen.bmj.com/site/about/guidelines.xhtml

17. International Institute for Population Sciences (IIPS) and ICF. National family health survey (NFHS-4), India, 2015–16: Odisha. Mumbai: IIPS; 2017.
18. Gourlay, A., G. Mshana, I. Birdthistle, G. Bulugu, B. Zaba, and M. Urassa. 2014. "Using vignettes in qualitative research to explore barriers and facilitating factors to the uptake of prevention of mother-to-child transmission services in rural Tanzania: A critical analysis," BMC Medical Research Methodology 14(1): 21.
19. Anonymous 2020
20. Anonymous 2021
21. Anonymous 2020
22. World Health Organization. (2018). Weekly iron and folic acid supplementation as an anaemia-prevention strategy in women and adolescent girls: lessons learnt from implementation of programmes among non-pregnant women of reproductive age. World Health Organization. https://apps.who.int/iris/handle/10665/274581. Accessed February 4, 2021.
23. Intensified National Iron Plus Initiative (I-NIPI). (2018). Ministry of Health and Family Welfare, Government of India. Anemia Mukt Bharat. https://anemiamuktbharat.info/wp-content/uploads/2019/09/Anemia-Mukt-Bharat-Brochure_English.pdfAccessed February 22, 2021.
24. Sanchis-Gomar F, Cortell-Ballester J, Pareja-Galeano H, Banfi G, Lippi G. Hemoglobin point-of-care testing: the HemoCue system. J Lab Automation. 2012;18(3):198–205.
25. Rogers, W.H. (1993). Regression standard errors in clustered samples. Stata Tech Bull; 13 (Reprinted in Stata Technical Bulletin Reprints, 3: 88–94): 19-23.
26. Viswanath, K., & Kreuter, M. W. (2007). Health disparities, communication inequalities, and eHealth. *American journal of preventive medicine*, *32*(5), S131-S133.
27. Donohue, G. A., Tichenor, P. J., & Olien, C. N. (1975). Mass media and the knowledge gap: A hypothesis reconsidered. *Communication research*, *2*(1), 3-23.
28. Viswanath, K., & Finnegan Jr, J. R. (1996). The knowledge gap hypothesis: Twenty-five years later. *Annals of the International Communication Association*, *19*(1), 187-228.
29. Girma, E., Gebretsadik, L. A., Kaufman, M. R., Rimal, R. N., Morankar, S. N., & Limaye, R. J. (2014). Stigma against people with HIV/AIDS in rural Ethiopia, 2005 to 2011: signs and predictors of improvement. AIDS and Behavior, 18(6), 1046-1053.
30. Rimal, R. N. (2000). Closing the knowledge-behavior gap in health promotion: The mediating role of self-efficacy. *Health communication*, *12*(3), 219-237.
31. Rimal, R. N., Flora, J. A., & Schooler, C. (1999). Achieving improvements in overall health orientation: Effects of campaign exposure, information seeking, and health media use. Communication Research, 26(3), 322-348.

For peer review only - http://bmjopen.bmj.com/site/about/guidelines.xhtml

CONSORT 2010 checklist of information to include when reporting a randomised trial*

Section/Topic	Item No	Checklist item	Reported on page No
**Title and abstract**			
	1a	Identification as a randomised trial in the title	1
	1b	Structured summary of trial design, methods, results, and conclusions (for specific guidance see CONSORT for abstracts)	1-2
**Introduction**			
Background and objectives	2a	Scientific background and explanation of rationale	2-3
	2b	Specific objectives or hypotheses	3-4
**Methods**			
Trial design	3a	Description of trial design (such as parallel, factorial) including allocation ratio	6-7
	3b	Important changes to methods after trial commencement (such as eligibility criteria), with reasons	n/a
Participants	4a	Eligibility criteria for participants	7-8
	4b	Settings and locations where the data were collected	4
Interventions	5	The interventions for each group with sufficient details to allow replication, including how and when they were actually administered	reported in protocol paper which is cited in text
Outcomes	6a	Completely defined pre-specified primary and secondary outcome measures, including how and when they were assessed	8-9
	6b	Any changes to trial outcomes after the trial commenced, with reasons	n/a
Sample size	7a	How sample size was determined	reported in protocol paper which is cited in text
	7b	When applicable, explanation of any interim analyses and stopping guidelines	n/a
Randomisation:			
Sequence generation	8a	Method used to generate the random allocation sequence	6-7
	8b	Type of randomisation; details of any restriction (such as blocking and block size)	6-7
Allocation	9	Mechanism used to implement the random allocation sequence (such as sequentially numbered containers),	6-7

For peer review only - http://bmjopen.bmj.com/site/about/guidelines.xhtml

concealment mechanism		describing any steps taken to conceal the sequence until interventions were assigned	
Implementation	10	Who generated the random allocation sequence, who enrolled participants, and who assigned participants to interventions	reported in protocol paper which is cited in text
Blinding	11a	If done, who was blinded after assignment to interventions (for example, participants, care providers, those assessing outcomes) and how	reported in protocol paper which is cited in text
	11b	If relevant, description of the similarity of interventions	reported in protocol paper which is cited in text
Statistical methods	12a	Statistical methods used to compare groups for primary and secondary outcomes	10-11
	12b	Methods for additional analyses, such as subgroup analyses and adjusted analyses	10-11
**Results**			
Participant flow (a diagram is strongly recommended)	13a	For each group, the numbers of participants who were randomly assigned, received intended treatment, and were analysed for the primary outcome	26
	13b	For each group, losses and exclusions after randomisation, together with reasons	26
Recruitment	14a	Dates defining the periods of recruitment and follow-up	reported in protocol paper which is cited in text
	14b	Why the trial ended or was stopped	n/a
Baseline data	15	A table showing baseline demographic and clinical characteristics for each group	21
Numbers analysed	16	For each group, number of participants (denominator) included in each analysis and whether the analysis was by original assigned groups	21
Outcomes and estimation	17a	For each primary and secondary outcome, results for each group, and the estimated effect size and its precision (such as 95% confidence interval)	11-13; 21-23
	17b	For binary outcomes, presentation of both absolute and relative effect sizes is recommended	11-13; 21-23
Ancillary analyses	18	Results of any other analyses performed, including subgroup analyses and adjusted analyses, distinguishing pre-specified from exploratory	11-13; 21-23

For peer review only

For peer review only - http://bmjopen.bmj.com/site/about/guidelines.xhtml

Harms	19	All important harms or unintended effects in each group (for specific guidance see CONSORT for harms)	n/a
**Discussion**			
Limitations	20	Trial limitations, addressing sources of potential bias, imprecision, and, if relevant, multiplicity of analyses	17
Generalisability	21	Generalisability (external validity, applicability) of the trial findings	14-17
Interpretation	22	Interpretation consistent with results, balancing benefits and harms, and considering other relevant evidence	13-17
**Other information**			
Registration	23	Registration number and name of trial registry	2
Protocol	24	Where the full trial protocol can be accessed, if available	6
Funding	25	Sources of funding and other support (such as supply of drugs), role of funders	2

*We strongly recommend reading this statement in conjunction with the CONSORT 2010 Explanation and Elaboration for important clarifications on all the items. If relevant, we also recommend reading CONSORT extensions for cluster randomised trials, non-inferiority and equivalence trials, non-pharmacological treatments, herbal interventions, and pragmatic trials. Additional extensions are forthcoming: for those and for up to date references relevant to this checklist, see www.consort-statement.org.

For peer review only

BMJ Open

How does a social norms-based intervention affect behavior change? Interim Findings from a cluster randomized controlled trial in Odisha, India

Journal:	*BMJ Open*
Manuscript ID	bmjopen-2021-053152.R1
Article Type:	Original research
Date Submitted by the Author:	22-Mar-2022
Complete List of Authors:	Sedlander, Erica; University of California San Francisco<br>Pant, Ichhya; George Washington University School of Public Health and Health Services<br>Bingenheimer, Jeffrey ; George Washington University Department of Health Policy<br>Yilma, Hagere; Boston University<br>Patro, Lipika; IPE Consulting<br>Mohanty, Satya; DCOR Consulting<br>Ganjoo, Rohini; The George Washington University<br>Rimal , Rajiv; Johns Hopkins University Bloomberg School of Public Health
**Primary Subject Heading**:	Global health
Secondary Subject Heading:	Public health, Evidence based practice
Keywords:	PUBLIC HEALTH, NUTRITION & DIETETICS, Public health < INFECTIOUS DISEASES

SCHOLARONE™
Manuscripts

I, the Submitting Author has the right to grant and does grant on behalf of all authors of the Work (as defined in the below author licence), an exclusive licence and/or a non-exclusive licence for contributions from authors who are: i) UK Crown employees; ii) where BMJ has agreed a CC-BY licence shall apply, and/or iii) in accordance with the terms applicable for US Federal Government officers or employees acting as part of their official duties; on a worldwide, perpetual, irrevocable, royalty-free basis to BMJ Publishing Group Ltd ("BMJ") its licensees and where the relevant Journal is co-owned by BMJ to the co-owners of the Journal, to publish the Work in this journal and any other BMJ products and to exploit all rights, as set out in our licence.

The Submitting Author accepts and understands that any supply made under these terms is made by BMJ to the Submitting Author unless you are acting as an employee on behalf of your employer or a postgraduate student of an affiliated institution which is paying any applicable article publishing charge ("APC") for Open Access articles. Where the Submitting Author wishes to make the Work available on an Open Access basis (and intends to pay the relevant APC), the terms of reuse of such Open Access shall be governed by a Creative Commons licence – details of these licences and which Creative Commons licence will apply to this Work are set out in our licence referred to above.

Other than as permitted in any relevant BMJ Author's Self Archiving Policies, I confirm this Work has not been accepted for publication elsewhere, is not being considered for publication elsewhere and does not duplicate material already published. I confirm all authors consent to publication of this Work and authorise the granting of this licence.

For peer review only

Title: How does a social norms-based intervention affect behavior change? Interim findings from a cluster randomized controlled trial in Odisha, India

Authors: Erica Sedlander, DrPH, MPH[1], Ichhya Pant, DrPH (c), MPH[2], Jeffrey Bart Bingenheimer, PhD,[2] Hagere Yilma, PhD (c), MPH[3], Lipika Patro,[4] Satya Mohanty, MS,[5] Rohini Ganjoo, PhD[6], Rajiv N. Rimal, PhD[2, 7]

[1] The University of California San Francisco, Department of Social and Behavioral Sciences
[2]The George Washington University, Milken Institute School of Public Health, Department of Prevention and Community Health
[3]Boston University, Sargent College, Department of Health Sciences
[4] IPE Global Limited
[5] DCOR Consulting
[6] The George Washington University, School of Medicine and Health Sciences, Department of Integrated Health Sciences
[7]Johns Hopkins University, Department of Health Behavior and Society

Erica Sedlander: (corresponding author)
University of California San Francisco, Department of Social and Behavioral Sciences
esedlander@gwmail.gwu.edu

Erica Sedlander: esedlander@gwmail.gwu.edu
Ichhya Pant: ipant@email.gwu.edu
Jeffrey Bart Bingenheimer: bartbing@gwu.edu
Hagere Yilma hyilma@gwmail.gwu.edu
Lipika Patro: lipika.patro@ipeckd.org
Satya Mohanty: satya.dcor@gmail.com
Rohini Ganjoo: rganjoo@email.gwu.edu
Rajiv N. Rimal: rimal@jhu.edu

For peer review only - http://bmjopen.bmj.com/site/about/guidelines.xhtml

Title: How does a social norms-based intervention affect behavior change? Interim findings from a cluster randomized controlled trial in Odisha, India

Abstract

Background: Behavior change interventions targeting social norms are burgeoning, but researchers have little guidance on what they look like, and which components affect behavior change. The Reduction in Anemia through Normative Innovations (RANI) project designed an intervention to increase iron folic acid consumption in Odisha, India.

Objective: This paper examines the effect of the intervention at midline to understand which components of the RANI intervention affect uptake.

Methods: Using a cluster randomized controlled design, we collected baseline data and midline data six months later from women of reproductive age in the control and treatment arms (n = 3,800) in Angul, Odisha, India. Using nested models, we analyzed data from three different intervention components, monthly community-based testing for anemia, participatory group education sessions, and

For peer review only - http://bmjopen.bmj.com/site/about/guidelines.xhtml

videos, to determine the extent to which exposure to each of these components accounted for the overall intervention effect on hemoglobin and self-reported IFA use.

Results: Overall, residing in a treatment as opposed to control village had little effect on midline hemoglobin, but increased the odds of taking supplements by 17 times. Exposure to each of the intervention components had a dose-response relationship with self-reported IFA use. These components, separately and together, accounted for most of the overall effect of treatment assignment on IFA use.

Conclusions: All intervention components increased iron supplement use to differing degrees of magnitude. It appears that a social norms-based approach can result in improving iron folic acid uptake, though improvements in hemoglobin counts were not yet discernible.

Keywords: social norms, behavior change, intervention, anemia

Strengths and limitations of this study

- The intervention is based on formative research, theory, and co-designed with input from community stakeholders
- Data comes from a large blinded cluster-randomized controlled trial
- Intervention components are qualitatively described and then quantitatively evaluated to decipher their individual effect on iron supplement use
- We rely on self-report for iron supplement use and image recall for participation in intervention activities

Introduction

Social norms, defined as informal rules of behavior considered acceptable in a group or society [1], are increasingly recognized as drivers of or barriers to behavior change. In a review of social norms-based interventions over the last three decades, more than half were published in the last decade [2]. Recent social norms interventions in low to middle income countries have focused on changing harmful behaviors, such as intimate partner violence [3-4], female genital cutting [5], and child marriage [6]. Past research shows that people are more likely to engage in a behavior when they believe many others also do so, and when there is a social expectation that they themselves should comply. In the anemia prevention context recent literature indicates a seemingly simple behavior, e.g., taking a weekly iron supplement, is embedded in social and cultural dynamics that dictate which health behaviors are appropriate [7-8].

The theory of normative social behavior (TNSB) highlights the critical role that social norms can play in influencing health behaviors and the circumstances under which they may do so [9-10]. According to the TNSB, the effects on behavior of descriptive norms (i.e., one's perceptions about others' behaviors) and injunctive norms (i.e., one's perceptions of social expectations regarding the behavior) may depend upon other factors at the individual, behavioral, and environmental level [11]. Factors that could either augment or attenuate the effect of social norms messaging on iron folic acid (IFA) use include perceptions that one is at risk of getting anemia, outcome expectations that taking IFA will result in health benefits like preventing anemia or reducing fatigue, and access to and availability of IFA. Therefore, interventions must aim to improve both social norms around a behavior along with potential moderators that may strengthen the relationship between social norms and behaviors. In a 2018 meta-analysis of social norms approaches to behavior change, Dempsey and authors found that the most effective social norms manipulations take place in one's own environment (e.g., a field trial), those that deliver messages in multiple formats, and those that target collectivist groups [12].

For peer review only - http://bmjopen.bmj.com/site/about/guidelines.xhtml

Many social norms-based interventions are often complex and resource intensive. To implement them well, program planners need to understand the social context, the social norms around the behavior of interest, and barriers and facilitators at multiple levels of the socio-ecological model [13,14]. Guided by the TNSB, the Reduction in Anemia through Normative Innovations (RANI) Project conducted formative research to delineate significant facilitators to and barriers of iron folic acid consumption in Odisha, India [8]. Findings from the formative work led to the design of the intervention, which included three components: (a) hands-on participatory learning modules conducted in small groups, (b) dissemination of short videos focusing on iron consumption norms, and (c) monthly hemoglobin testing for anemia, followed by public display of (anonymized) community results.

In this paper, we seek to determine which intervention components impact iron folic acid consumption six months later at midline. This knowledge allowed us to adapt the intervention implementation according to empirical findings. Since social and political realities on the ground cannot be fully predicted at the outset of any given field trial [15], we deemed this approach more preferable than simply implementing a static intervention based on *a priori* data. Thus, the primary goal of this paper is to examine the extent to which each intervention component contributed to the overall effects of the intervention. In addition, because the influence of the intervention may be different across subgroups, we also examine how susceptibility to intervention impact varied by age, caste, education, and communication activity. We hope that delineating the effects of each intervention component will provide guidance for future social norms-based intervention designs. As Davis et al. ([69], p. 2218) argue, "while the social norms approach is based in a rich theory, the theory does little to illuminate implementation details of interventions [16]."

Methods

For peer review only - http://bmjopen.bmj.com/site/about/guidelines.xhtml

This study was approved by the George Washington University Institutional Review Board (FWA00005945), Sigma Science and Research, an independent IRB located in New Delhi, India, and the Indian Council for Medical Research's Health Ministry's Screening Committee.

Patient and Public Involvement Statement

Key stakeholders from the community where the intervention was implemented participated in a two-day convening in Bhubaneswar, Odisha to co-design the intervention. Community health workers helped to implement the intervention and disseminate findings back to the community. Patients were not involved in the study – participants were living in the villages where the intervention took place.

Study Setting

Nestled in the eastern coast of India, the state of Odisha is predominantly rural. Most residents (95%) are Hindu, with 23% belonging to specific tribes and practicing tribal culture (NFHS 2015-2016). At approximately 2.1 children per woman, the total fertility rate sits fairly low. Our focal district, Angul, one of 30 in the state of Odisha, has almost 2,000 villages with a total population of just over 1.2 million (Government of Odisha, 2019). Men's literacy rate (87%) is higher than that of women's (70%). Almost a quarter of girls (22%) marry before age 18 and around half of married women of reproductive age use modern methods of family

For peer review only - http://bmjopen.bmj.com/site/about/guidelines.xhtml

planning [17].

Intervention Development

To develop the RANI intervention, we conducted formative research that examined social norms around IFA use. Between March and May 2018, we collected data from four villages in the two adjacent blocks (administrative units below the district) where the intervention took place (Kishorenagar and Athamalik). We conducted 16 focus groups and 21 individual interviews (n = 148), stratified by age and gender, with women of reproductive age, husbands, mothers-in-law, and key informants. To explore women's social norms within the focus groups, we used vignettes, short stories about theoretical characters that also live in a rural village in Angul, India [18]. Vignettes can also help uncover if social sanctions exist and unpack existing social norms. Four researchers, two from India and two from the United States, analyzed transcripts using NVivo v.12 to identify barriers and facilitators to IFA use.

We found that social norms and available services varied substantially for pregnant women, non-pregnant women, and adolescents. Specifically, we found that most participants believed only pregnant women and adolescents in school consume IFA (descriptive norms). Participants also stated that only pregnant women and those diagnosed with anemia should be taking IFA regularly (injunctive norms) and we found that frontline health workers only distributed IFA to pregnant women. Adolescents enrolled in school can also obtain them weekly [8].

Non-pregnant women (our sample for this paper) were not receiving IFA from frontline health workers. Indeed, barriers faced by non-pregnant women were significant: they needed to visit a health center, get tested for anemia, and then obtain the IFA if they were

For peer review only - http://bmjopen.bmj.com/site/about/guidelines.xhtml

diagnosed as anemic. We also found that risk perception was low, with most participants believing that only “a handful of women” in their community had anemia when, in reality, more than half of women are anemic. When anemia was referenced, we found that participants were primarily referring to severe anemia, not its mild or moderate forms [8].

Our findings also revealed that inequitable gender norms were an upstream barrier to women’s accessing and adhering to IFA supplements. Specifically, women prioritized their family’s health and well-being over their own, normalized fatigue as part of a woman’s plight, and given that they often do all of the household work while also working outside of the home, they lacked time (and often autonomy) to visit a health center on their own [19-20].

We used findings from the qualitative research, past literature on anemia reduction efforts, and the TNSB to design the RANI intervention. A three-day convening in Bhubaneswar, Odisha, was held where we invited frontline health workers from the community, anemia researchers, and program planners to co-design an effective social norms-based intervention. Finally, we used quantitative data collected at baseline to refine the intervention design. Specifically, we validated qualitative findings that very few non-pregnant women were taking IFA with a specific percentage (less than 3% of women) despite guidelines that all women should take them regularly to prevent anemia [21]. Therefore, we decided to focus more on injunctive norms messaging (that all women of reproductive age should take IFA) rather than descriptive norms messaging (that women are taking IFA) in the beginning of the intervention.

For peer review only - http://bmjopen.bmj.com/site/about/guidelines.xhtml

The RANI Project intervention comprises three main components, each tapping into social norms differently: participatory learning modules; RANI Comms (videos), and community hemoglobin testing. A full description of all RANI intervention activities including specifics about each component can be found in **Table 1**. All RANI project data is stored in an online data repository [22].

Study Design

Data for this study report interim midterm findings from the main trial of the RANI project [23]. The RANI project uses a cluster randomized controlled trial to evaluate the efficacy of a norms-based intervention to increase IFA use and reduce anemia in Odisha, India. The RANI project selected two blocks within the Angul district in the state of Odisha. We grouped contiguous villages in these blocks into clusters, which we then randomly assigned to either the treatment or control arm. Villages in the treatment arm were exposed to the RANI intervention, while villages in the control arm continued with "care-as-usual". We created clusters to minimize contamination; clusters were separated by either a natural buffer (i.e., mountain or river) or a village that was neither in the treatment nor in the control arm. This process resulted in a total of 89 clusters from 239 villages. We then segmented clusters by the proportion of caste/ethnic groups (in India they are called scheduled castes and scheduled tribes) and then selected 3 per stratum, for a total of 15 clusters per arm to be included in data analysis (which comprised 81 villages).

In this paper, we report results from the baseline and midterm assessment, which is a longitudinal study from both the treatment and control clusters. The response rate for the midline questionnaire was 96.2%. Interviewers visited homes up to three times and the primary reason for not taking the midline survey was not being home when the interviewers visited their house.

Participants

For peer review only - http://bmjopen.bmj.com/site/about/guidelines.xhtml

In each designated village, we first enumerated all households and then randomly selected households for data collection using proportion-to-size principles based on cluster population. From the selected home, one woman of reproductive age (between 15 and 49 years old) was chosen (randomly if more than one woman was eligible in the same home). Although our sample consisted of 3,953 participants, this paper restricts the sample to those who were not pregnant at the time of the midline survey (n = 3,800). We do so because the primary dependent variable, taking iron and folic acid tablets, has been heavily promoted among pregnant women by the Government of India. Pregnant women are also enrolled in the health system, where physicians or community health workers provide free IFA. This is not the case among non-pregnant women, who have not been targeted as IFA recipients on the ground despite WHO and Indian government recommendations [24-25]. The demographic profile of participants included in our analysis is shown in Table 2.

Procedure

The baseline data was collected in September 2019 and midline data was collected six months later in February 2020. Local data collectors obtained informed consent from all individual participants included in the study in the local language, Odiya. Participants under the age of 18 were required to obtain the written permission of one parent or legal guardian. Data collectors orally administered a one-on-one survey to all participants, which assessed demographic information, psychosocial factors, and anemia-related behaviors.

Inclusion Criteria

For peer review only - http://bmjopen.bmj.com/site/about/guidelines.xhtml

Women were eligible for inclusion in the study if they were between the ages of 15 and 49, spoke Odiya, lived in the data collection villages (either treatment or control), and did not plan to move in the next year (as this is a longitudinal study).

Measures

Dependent Variables. The RANI study was evaluating the efficacy of a norms-based intervention to increase IFA use and reduce anemia. Therefore, our study has two dependent variables: self-reported IFA use and objectively measured serum hemoglobin levels. We measured *IFA use at midline* using the interview question, "Have you ever eaten/taken an iron tablet or syrup." (The interviewer then held up the packet of IFA tablets for the interviewee to see). We coded this as a dichotomous variable, scored 1 if currently taking and 0 if not or did in the past but stopped now. We obtained *hemoglobin levels* from all participants at midline through point-of-care hemoglobin tests using a HemoCue photometer (in line with India's National Family Health Survey methodology). This instrument provides hemoglobin levels immediately and accurately [26].

Independent Variables (Exposure to the Intervention). We examined four independent variables. The first is a dichotomous indicator of *treatment assignment* that takes the value 1 for participants residing in intervention villages and 0 for those residing in control villages. The other three independent variables are participants' self-reports of exposure to different components of the RANI intervention. To measure *exposure to the participatory learning modules*, we took the sum of responses to six questions about how often participants had seen materials from these sessions. We used visual images from the sessions, with higher scores indicating more exposure or more frequent exposure (not seen = 0, seen once or twice = 1, and seen more than twice = 2). Each image came from a different participatory learning module. One question asked whether or not they had participated in any of the games that were also a

For peer review only - http://bmjopen.bmj.com/site/about/guidelines.xhtml

part of the RANI participatory group sessions (scored as No=0 and Yes=1). Less than 1% of participants (*n=28*) marked "don't know" (which was coded as missing). We coded exposure to participatory learning sessions as a continuous variable (range 0-11). We assessed participants' *own anemia testing* (exposure to the anemia testing component of the intervention) with the question, "How many times have you been tested for anemia as part of the RANI intervention in the last six months?" Response options ranged from 0 (never) to 4 (more than 3 times). We also coded testing for anemia as a continuous variable (range 0-4). We measured *exposure to the RANI Comm videos* as the sum of responses to four questions about which of the four videos they had watched. Interviewers shared an image from each video and a brief description of the story plot. Responses were treated as a continuous variable and summed across the four videos for a range from 0 to 4. We assessed *communicating with others about intervention components* with three separate questions: "Talked about blood tests when talking about RANI to friends or family;" "Talked about videos when talking about RANI to friends or family;" and "Talked about RANI meetings (group education modules) when talking about RANI to friends or family." For each, an affirmative response was coded 1 and a negative response was coded 0.

Control Variables. We asked respondents their *age*, highest completed level of *education*, and whether they belonged to a *scheduled tribe*. *IFA use at baseline* was assessed exactly as described above for midline. We asked respondents about the *number of children* they had and whether or not they were *breastfeeding*. Additionally, to understand if participants had been exposed to another intervention that was not affiliated with RANI (to avoid contamination), we asked participants, "Did you hear anything about nutrition or iron tablets from the Swabhimaan or any other program?" We coded this as a dichotomous variable (No or don't know = 0 and Yes = 1).

For peer review only - http://bmjopen.bmj.com/site/about/guidelines.xhtml

Statistical Analysis

We conducted our analyses in three steps. First, we calculated frequencies and descriptive statistics of all key analytic variables by treatment and control arm and obtained p-values testing the null hypothesis of no difference between the two arms via linear and logistic for continuous and categorical variables, respectively. These analyses covered several socioeconomic and health-related background variables; amount of exposure to each of the three intervention components between baseline and midline; and hemoglobin levels and self-reported IFA use at midline. The latter provided unadjusted intention-to-treat estimates of the overall RANI treatment effect on these primary endpoints at midline.

Second, we ran linear regressions to examine if exposure to each intervention component varied by select sociodemographic factors: age (32 years or older versus below 32 years old), belonging to a schedule caste or not, and education (completed primary school or not). These analyses were limited to residents of clusters assigned to the RANI intervention arm.

Third, we ran a series of regression models to examine how each intervention component individually and additively affected hemoglobin levels (linear) and IFA use (logistic) at midline and accounted for the overall effect of treatment assignment. For each outcome, Model 1 includes only control variables (age, education, currently breastfeeding, number of children, whether they belong to a scheduled caste or tribe, knowing anemia status at baseline, IFA use at baseline, and whether the participant reported exposure to a non-RANI intervention) and RANI treatment assignment. The coefficients on RANI treatment assignment in these models represent adjusted intention-to-treat estimates for each outcome. In Model 2, we add a set of dummy variables representing levels of exposure to the first RANI intervention component, group education sessions, with zero sessions as the reference category. The dummy variable

For peer review only - http://bmjopen.bmj.com/site/about/guidelines.xhtml

specification provides flexibility in the nature of the relationship between number of group education sessions attended and the two outcome variables. The coefficients on the dummy variables in this model represent how midline hemoglobin and self-reported IFA use vary in relation to number of group education sessions attended. Moreover, comparison of the coefficient on the RANI treatment assignment variable in this model to the analogous coefficient in Model 1 provides insight into the extent to which group education sessions contributed to the overall effect of RANI. The next two models are similar to Model 2 but using hemoglobin testing (Model 3) and viewing of RANI Comm videos (Model 4) instead of group education session attendance. Finally, Model 5 includes all three intervention components. The coefficients on the intervention component dummy variables in this model provide some insight into the unique contribution of each component, independent of the other two. And comparison of the coefficient on the RANI treatment assignment dummy variable in this model to the corresponding coefficient from Model 1 represents the extent to which the three intervention components jointly contributed to the overall RANI intervention effect. We used Stata version 16 to conduct all analyses, with Huber-White clustered standard errors [27] to account for the sampling and cluster randomization design.

Results

Description of the sample included in our study is shown in Panel A of Table 2. Average age was 31 years old and between a quarter and a third of participants were a part of the tribal population. On average, participants completed primary school (6 years of education). Participants in both treatment and control arms had on average more than one child and fewer than two. About 21% of women in both arms were currently breastfeeding. The only statistically significant difference between the treatment and control arms was age. Women in the treatment arm were 31 years old versus 30 years old in the control arm ($p < .05$). All other demographics were

For peer review only - http://bmjopen.bmj.com/site/about/guidelines.xhtml

not statistically different by treatment and control. Panel B of Table 2 shows that exposure to non-RANI interventions was low in both study arms.

Crude estimates of the effects of the RANI intervention at midline are shown in Panel C of Table 2. Participants from the treatment arm reported more IFA use at midline (32%) compared to the control arm (3%) (p = < .001). However, hemoglobin levels were not statistically different in the two arms (11.7% versus 11.5%) (p =.28) (see Appendix Table 1). Panel D of Table 2 shows that exposure to each of the intervention components was widespread among participants in the treatment arm and low among participants in the control arm. Specifically, 88% of participants in the treatment arm compared to only 12% in the control arm reported that they attended at least one group educational session (p = <.001). Over eighty percent of participants in the treatment arm reported that they had been tested at least once as part of the intervention compared to 0% in the control arm (p = <.001). Similarly, 80% of women in the treatment arm reported that they watched one or more RANI Comm videos, compared to less than 1% in the control arm (p = <.001). Lastly, exposure to interventions other than RANI was minimal across both the treatment and control arms (Panel B, 1.2% and 2.1%, respectively).

Table 3 shows that less educated women attended more educational sessions (4.77 compared to 4.46; p = < .05) but more educated women had a higher average number of anemia tests(1.32 compared to 1.22; p = < .05). Table 3 also shows that neither age nor caste or tribal background was associated with exposure to the intervention.

As there was no crude difference between the treatment and control arms in hemoglobin level at midline (see Panel C of Table 2), we included the analyses linking exposure to specific intervention components to hemoglobin levels as an appendix (See Appendix

For peer review only - http://bmjopen.bmj.com/site/about/guidelines.xhtml

Table 1). However, nested logistic regression models predicting self-reported IFA use as a function of study arm and exposure to each of the intervention components are presented in Table 4. As seen in Model 1, after controlling for demographic variables and IFA use at baseline, simply being in the treatment arm increased a woman's odds of taking IFA by more than 16 times (OR = 16.94; p = < .001). In Model 2, we added exposure to the group education sessions and found that each session that a woman attended significantly increased her odds of taking IFA . Model 3 suggests that each additional test for anemia also significantly increased the odds of taking IFA. Model 4 similarly shows that for each RANI Comm video that a woman watched increased her odds of taking IFA increased. Model 5 with all intervention components included shows that while all effect sizes drop (including the effect of RANI treatment assignment), each component still had a significant effect on IFA use This indicates that each component explains a unique part of the variance in current use of IFA.

Discussion

In this paper we present findings from the midline interim assessment of a multicomponent behavioral intervention intended to increase IFA use and decrease anemia among women of reproductive age in Odisha, India. We find strong evidence that the intervention increased the prevalence of self-reported IFA use among non-pregnant women, but no evidence of an effect of the intervention on average hemoglobin levels in that group. Self-reported exposure to all three intervention components was high among women in the treatment arm, which did not vary substantially by age or membership in a scheduled caste or tribe. However, exposure to anemia testing was higher among more educated women and exposure to group sessions was higher among less educated women.

For peer review only - http://bmjopen.bmj.com/site/about/guidelines.xhtml

Self-reported exposure to all three intervention components was positively associated with IFA use, and these exposures accounted for most but not all the overall effect of the RANI intervention on IFA use among non-pregnant women.

Given that we saw very little difference in exposure to the intervention components by age, education or caste, it appears that the RANI Project's outreach efforts did not disproportionately favor those with higher privilege in society. Indeed, even though exposure to the intervention did not differ by tribal status or age, in our multivariate models we found some evidence that those with lesser education had higher IFA uptake. We view this finding rather optimistically, given that interventions have the potential for exacerbating existing differences in society along access and socioeconomic lines [28], as has been documented by the literature on the knowledge gap hypothesis [29-30].

We saw strong evidence that testing women for anemia (through finger-prick hemoglobin counts) was associated with IFA uptake: those who got tested more often were more likely to consume IFA tablets. The reasons for this are not known precisely, but we do have a few explanations. First, it could be that testing revealed to women the low hemoglobin counts, which spurred them to act to improve their scores. Our formative assessment had revealed that feeling weak was often a part of women's self-identity, with women often believing that fatigue was par for the course [19] and that this was part of the gendered norms for women [20].We suspect that, in contrast, an objective measure of iron in the blood, observed through hemoglobin testing, quantified and thus provided precision to a phenomenon woman had come to accept as a vague notion of fatigue. We found that testing was highly demanded and motivational. Indeed, our process evaluation data indicate that, despite a high (60%) coverage rate of anemia testing for women in our

intervention catchment areas, we have been unable to meet the demand for testing and repeat testing. If were able to meet the demand, the impact of testing may have been even higher.

A second reason why testing may have motivated women to consume IFA tablets pertains to observational learning around social norms. Due to test results being displayed publicly in the community, and most educational sessions linked the test results with IFA consumption, women likely came to link their consumption behaviors with hemoglobin readings – not only for themselves but also for what they had observed among others in their community, where hemoglobin testing was a monthly event. These events were so popular that, in some villages, the waiting list for testing ran up to 90 names (we were unable to accommodate more than 15 women per village per month, given the study design and resources). This public display of IFA testing and hemoglobin results also normalized women prioritizing their own health potentially shifting gender norms that solely focus on pregnant women's health or the health of the family. Although these underlying reasons are somewhat speculative, they do point to the need to study more precisely the link between testing and IFA consumption.

Each educational participatory learning session that women attended significantly increased IFA use. These sessions were designed primarily to raise awareness and improve knowledge about anemia, iron-rich foods, and diet diversity. We also included discussions about gender roles, how eating order in the home disfavors women's health, and the need to remain strong to be able to take care of others. We have few reasons to believe that knowledge in these domains would directly translate into behaviors. However, a significant body of work demonstrates that knowledge about health is a necessary, though not sufficient, condition for behavior

For peer review only - http://bmjopen.bmj.com/site/about/guidelines.xhtml

change [31 - 32] and that people often hold knowledge in abeyance, to be acted upon at the appropriate time [33]. From this perspective, it seems that educational sessions may have played an important role.

Finally, we found that exposure to the communication videos was also significantly associated with increased IFA uptake. We suspect that the underlying reason for this finding pertains to another normative component of the intervention. The four videos we developed targeted different audiences, including adolescents, husbands, mothers-in-law, and women of reproductive age. As an explicit campaign strategy, each video was shown to each of the parties, including those who were not the explicit target audience for the particular video. This was done so that each group came to understand that the other groups were also being targeted by the intervention. So, for example, adolescent girls saw that there were videos that also addressed men and older women. Similarly, men saw that the videos targeted other men and other women. The overall strategy was to communicate the message that the entire community was engaged in the task of reducing anemia.

In this paper, we assessed how each of the intervention components affected IFA use. This helps elucidate where this intervention should focus for the remaining trial, provides a clear picture of dose response, and highlights where other social norms program implementors may want to focus their efforts. In another midline paper, we assessed the effect of social norms on IFA use and found that changes in descriptive and collective norms (but not injunctive norms) were associated with changes in self-reported IFA use [34].

It is also important to discuss the lack of an effect of the RANI intervention on hemoglobin use despite an increase in self-reported IFA use. One plausible explanation of these disparate findings is that the increase in IFA use among members of the

For peer review only - http://bmjopen.bmj.com/site/about/guidelines.xhtml

treatment arm is real but is not sufficient in magnitude or duration to produce a corresponding increase in hemoglobin levels. Alternatively, it could be attributable to differential misreporting of IFA use among treatment group members, a form of courtesy or social desirability bias. Findings from the end line data collection, planned to take place at the end of the project, may help to adjudicate between these two explanations.

Of course, our study is not without limitations. First and foremost, while treatment assignment is objectively measured on the basis of project administrative data, exposure to the three intervention components is self-reported and may be subject to some level of misreporting. Furthermore, it is possible that women who participate in the intervention may be more motivated to change behavior in general and are already more inclined to take IFA. Therefore, Table 4 could overstate the effects on IFA use of exposure to the intervention components, as well as the contributions of those components to the overall RANI effect. However, IFA use is still a result of participating in the RANI intervention, as participation led to IFA use so we may have simply captured women who were farther along in their readiness to change. Additionally, although our study used a representative sample of areas and women in our focal areas, findings may not be generalizable outside of that setting.

Despite these limitations, our study has strengths: the intervention is based on formative research; it is co-designed by stakeholders from the area; we use a theoretically driven and adaptive approach; and the study design itself is robust, with an underlying randomization to experimental arms.

Conclusion

For peer review only - http://bmjopen.bmj.com/site/about/guidelines.xhtml

Our findings show that a social norms-based intervention can be successful in increasing IFA use. They also demonstrate that unique intervention components separately and altogether impact this success. All three components appear to contribute something to the overall effect of the intervention. While all three intervention components tapped into social norms messaging, hemoglobin testing provided individual health information, village level health information, a comparison to other villages, and changes in health information over time. This multi-level component, coupled with the other two components, may help women reach the tipping point to take and adhere to IFA. While IFA use shows promise, hemoglobin levels may need more time to show significant changes, especially among non-pregnant women who were not taking IFA and who only take it weekly. End line results will elucidate more information about the full RANI intervention effects.

For peer review only - http://bmjopen.bmj.com/site/about/guidelines.xhtml

Trial Registration
This trial was registered with Clinical Trial Registry- India (CTRI) (CTRI/2018/10/016186) on 29 October 2018.

Funding
This work was supported by a grant from The Bill and Melinda Gates Foundation (OPP1182519) to the George Washington University, Rajiv N. Rimal, principal investigator. The funders had no role in study design, data collection and analysis, decision to publish, or preparation of the manuscript.

Author Contribution Statement
ES: Conceptualization, methodology, analysis, writing – original draft
IP: Conceptualization, methodology, writing – review & editing
JB: Methodology, writing – review & editing
HY: Methodology, data curation, writing – review & editing
LP: Project administration, supervision, writing – review & editing
SM: Project administration, supervision, writing – review & editing
RG: writing – review & editing
RR: Conceptualization, methodology, supervision, funding acquisition, writing – review & editing

The authors have no competing interests.

Data sharing statement:

Data used for this study can be found in our online data repository: GW Scholarspace (https://scholarspace.library.gwu.edu/) and can also be requested from the first author.

Table 1. Description of RANI intervention activities

**Group participatory learning sessions**

For peer review only - http://bmjopen.bmj.com/site/about/guidelines.xhtml

We developed ten one-hour-long group participatory learning modules on various topics related to anemia prevention, including iron folic acid supplementation, diet diversity, and social norms/gender norms that may impact a woman's ability to take iron supplements. These monthly group participatory learning sessions are delivered through in-person activities and games. Women and their social networks (e.g., husbands and mothers-in-law) were all invited to participate so that women and those important to her are being exposed to the same messaging. We covered four participatory learning modules before midline data collection. Testing sessions lasted for an hour as community/group testing was followed up by demonstration of results and behavioral nudges for improving their Hb count.
**RANI Comm videos**<br>We created four RANI Comm videos in the local language, Odiya, with local residents as actors. These videos highlighted the stories of women overcoming barriers related to IFA consumption and other social norms prevalent in the area. The videos were shown on smartphones and tablets to both individuals and small groups. The videos targeted various audiences (pregnant women, non-pregnant women, husbands, and mothers-in-law) and addressed barriers and facilitators to IFA use that we identified in the formative research

For peer review only

followed by group discussion sessions. Descriptive and injunctive norms messages around IFA consumption were included in each storyline. An example of injunctive norms messaging in the storyline is a mother-in-law expressing that her daughter-in-law should be taking care of herself too, not just looking after the family, and should be taking weekly IFA to avoid anemia even if she is not pregnant. A new video was rolled out every 2-3 months. The viewing time for one video was approximately 15 minutes. Even though videos were 3-4 minutes long, the pre and post viewing discussion took an additional 10 minutes. Participants could have seen the same video more than once.	
**Community based hemoglobin testing**<br>We also conducted monthly anemia testing of the women using a digital Hemocue meter. These instant results are shared at the individual, group, and village levels with the help of blood shaped cards (different colors indicating anemia severity) and infographics appropriate for a low literacy population. Monthly community-based testing was followed by a discussion about trends in anemia and village-level comparisons (based on the hemoglobin readings) with neighboring communities at both the individual and community levels. This provides ipsative feedback (information people receive about their ongoing progress over time), normative	

For peer review only

feedback (information about the particular individual's achievements relative to those of her social peer), and aspirational feedback (comparisons people make between their current state of affairs and the goals they may have set for themselves).

* Community Facilitators of the RANI team delivered the intervention. However, IFA tablets were provided by the community health workers. RANI started off using key influencers in the village like front line health workers to gather people for the intervention. However, as our facilitators became familiar with the community, we also accepted RANI volunteers (women from the community) who facilitated this. Some of the interventions were delivered at the household level also.

Table 2. Description of Participants by Study Arm

	Treatment (n=1874)	Control (n=1867)	p-value
Demographic			
Age (years)	31.2	30.5	0.022
Part of tribal pop	23.6%	31.7%	0.267
Education (years)	6.07	6.17	0.749
Breastfeeding at baseline	21.4%	21.7%	0.836
Number of children at baseline	1.69	1.68	0.851
Exposure to non-RANI interventions	1.2%	2.1%	0.283
Dependent variables			
IFA use at midline	32.0%	3.0%	<0.001
Hemoglobin at midline (g/dl)	11.66	11.52	0.281
Exposure to RANI intervention components			
Number of group education sessions attended	4.60	0.23	<0.001

For peer review only - http://bmjopen.bmj.com/site/about/guidelines.xhtml

Attended at least one group education session	88.6%	12.1%	<0.001
Number of hemoglobin tests undergone	1.28	0.00	<0.001
Tested for hemoglobin at least once	81.4%	0.0%	<0.001
Number of RANI Comm videos views	2.46	0.00	<0.001
Viewed at least one RANI Comm video	79.8%	0.2%	<0.001

Notes: *T*-tests compare demographic differences between treatment versus control arms. Significant cells are in bold. IFA = iron folic acid. *T*-tests compare IFA use and exposure to different intervention components by treatment and control arms. Significant cells are in bold. The other non-RANI related intervention includes anemia related education as one component that may have had some overlap with our intervention. We included this as a control in our models to ensure that any changes in behavior are a result of our intervention alone.

Table 3. Exposure to intervention components by age, education, and caste (treatment arm participants)

	Average number of group educational sessions attended	Average number of hemoglobin tests undergone	Average number pf RANI Comm videos seen
Age			
Less than 32 years	4.48 (SD: 2.82)	1.33 (SD: 1.04)	2.41 (SD: 1.55)
32 years or more	4.74 (SD: 2.95)	1.22 (SD: 0.95)	2.53 (SD: 1.55)
p-value	0.219	0.056	0.119
Education			
Up to completed primary	4.77 (SD: 2.88)	1.22 (0.93)	2.55 (SD: 1.55)
More than completed prim	4.46 (SD: 2.88)	1.32 (1.05)	2.40 (SD: 1.55)
p-value	0.040	0.033	0.132
Scheduled Tribe or Caste			
Yes	4.54 (SD: 2.87)	1.28 (SD: 1.00)	2.44 (SD: 1.56)
No	4.78 (SD: 2.90)	1.29 (SD: 1.01)	2.55 (SD: 1.52)
p-value	0.429	0.899	0.407

For peer review only - http://bmjopen.bmj.com/site/about/guidelines.xhtml

For peer review only

*SD = Standard deviations

Table 4. Adjusted odds ratios from logistic regression models predicting self-reported IFA use at midline as a function of treatment assignment, exposure to three intervention components, and control variables

	Model 1	Model 2	Model 3	Model 4	Model 5
RANI Intervention Overall	16.94***	5.24***	3.62***	5.16***	2.25*
Group education sessions					
1 (vs. 0)		1.50			1.17
2 (vs. 0)		2.89***			1.71*
3 (vs. 0)		2.94***			1.37
4 (vs. 0)		3.05***			1.30
5 (vs. 0)		3.80***			1.43
6 (vs. 0)		5.37***			1.76*
7 (vs. 0)		5.39***			1.39
8 (vs. 0)		8.53***			2.41*
9 (vs. 0)		8.16***			2.30**
10 (vs. 0)		9.23***			2.23*
11 (vs. 0)		11.97***			2.81**
Anemia testing					
1 (vs. 0)			4.91***		3.07***
2 (vs. 0)			6.93***		3.72***
3 (vs. 0)			11.94***		5.65***
4+ (vs. 0)			12.91***		4.90***
RANI Comm videos					
1 (vs. 0)				2.30***	1.44
2 (vs. 0)				2.85***	1.59
3 (vs. 0)				3.57***	1.72*
4+ (vs. 0)				5.66***	2.39***
Control Variables					
Age	0.99	0.99	1.00	0.99	0.99
Education	0.98	0.97	0.97	0.98	0.97
Breastfeeding	1.35*	1.37*	1.41*	1.41**	1.43**

For peer review only - http://bmjopen.bmj.com/site/about/guidelines.xhtml

Number of children	0.99	0.96	0.96	0.97	0.95
Caste/Tribe	1.31*	1.26	1.31*	1.28	1.26
Knows anemia status at baseline	1.00	1.00	1.00	1.00	1.00
Baseline IFA use	2.59***	2.94***	3.30***	2.87***	3.36***
Non-RANI intervention exposure	1.85	1.28	1.57	1.59	1.36
Pseudo R2	0.20	0.24	0.24	0.23	0.26

Notes: *$p < .05$, **$p < .01$, ***$p < .00$

Ethical Approval Statement

This study was approved by the George Washington University Institutional Review Board (FWA00005945), Sigma Science and Research, an independent IRB located in New Delhi, India, and the Indian Council for Medical Research's Health Ministry's Screening Committee.

For peer review only

References

1. Reno, R. R., Cialdini, R. B., & Kallgren, C. A. (1993). The trans situational influence of social norms. *Journal of personality and social psychology*, *64*(1), 104.
2. Yemin, P., Fei, M., Lahlou, S., & Levy, S. (2019). Using social norms to change behavior and increase sustainability in the real world: A systematic review of the literature. Sustainability, 11(20), 5847.
3. Simone Francis, Prabin Nanicha Shrestha, Binita Shrestha, Gemma Ferguson , Brian Batayeh, Monique Hennink & Cari Jo Clark (2020): The Influence of Organised Diffusion on Social Norms Change: Addressing Intimate Partner Violence in Nepal, Global Public Health, DOI: 10.1080/17441692.2020.1845767
4. Clark, C. J., Bergenfeld, I., Batayeh, B., Ferguson, G., Shrestha, B., & Shrestha, P. N. (2019). Intimate partner violence in Nepal: Change starts at home study, what works to prevent violence: A global programme to prevent violence against women and girls evidence brief. South Africa Medical Research Council.
5. Shell-Duncan, B., Wander, K., Hernlund, Y., & Moreau, A. (2011). Dynamics of change in the practice of female genital cutting in Senegambia: testing predictions of social convention theory. Social science & medicine (1982), 73(8), 1275–1283. https://doi.org/10.1016/j.socscimed.2011.07.022
6. Vaitla, Bapu, Alice Taylor, Julia Van Horn, and Ben Cislaghi. 2017. Social Norms and Girls' Well-Being: Linking Theory and Practice. Washington, D.C.: Data2X.
7. Nagata, J. M., Gatti, L. R., & Barg, F. K. (2012). Social determinants of iron supplementation among women of reproductive age: a systematic review of qualitative data. Maternal & child nutrition, 8(1), 1-18.
8. Sedlander, E., Long, M., Mohanty, S., Munjral, A., Bingenheimer, J., Yilma, H., Rimal, R. N. (2020). Moving Beyond Individual Barriers and Identifying Multi-level Strategies to Address Anemia in Rural India. BMC Public Health, 20:457.
9. Rimal, R. N., Lapinski, M. K., Cook, R. J., & Real, K. (2005). Moving toward a theory of normative influences: How perceived benefits and similarity moderate the impact of descriptive norms on behaviors. Journal of health communication, 10(5), 433-450.
10. Rimal, R. N., & Real, K. (2005). How behaviors are influenced by perceived norms: A test of the theory of normative social behavior. Communication research, 32(3), 389-414.
11. Chung, A., and Rimal, R. N. (2016). Social norms: a review. *Rev. Commun. Res.* 4, 1–29. doi: 10.12840/issn.2255-4165.2016.04.01.008
12. Dempsey, R., McAlaney, J., Bewick, B. (2018). A Critical Appraisal of the Social Norms Approach as an Interventional Strategy for Health-Related Behavior and Attitude Change. Frontiers in Psychology, 6. https://doi.org/10.3389/fpsyg.2018.02180
13. Cislaghi, B., Nkwi, P., Mackie, G & Shakya, H. (2020). Why context matters for social norms interventions: The case of child marriage in Cameroon, Global Public Health, 15:4, 532-543, DOI: 10.1080/17441692.2019.1704818
14. Edberg, M., & Krieger, L. (2020). Recontextualizing the social norms construct as applied to health promotion.. *SSM-Population Health, 10* (). http://dx.doi.org/10.1016/j.ssmph.2020.100560
15. Pant, I., Patro, L., Sedlander, E., Gupta, K., Chandarana, S., Rimal, R. Monitor to Innovate with Feedback Loops: Process Evaluation Protocol for an Anemia Prevention Intervention. Study Protocol. Research Square. 10.21203/rs.3.rs-389608/v1

For peer review only - http://bmjopen.bmj.com/site/about/guidelines.xhtml

16. Davis, C.A.; Heiman, J.R.; Menczer, F. A Role for Network Science in Social Norms Intervention. Procedia Comput. Sci. 2015, 51, 2217–2226.
17. International Institute for Population Sciences (IIPS) and ICF. National family health survey (NFHS-4), India, 2015–16: Odisha. Mumbai: IIPS; 2017
18. Gourlay, A., G. Mshana, I. Birdthistle, G. Bulugu, B. Zaba, and M. Urassa. 2014. "Using vignettes in qualitative research to explore barriers and facilitating factors to the uptake of prevention of mother-to-child transmission services in rural Tanzania: A critical analysis," BMC Medical Research Methodology 14(1): 21.
19. Yilma, H., Sedlander, E., Rimal, R. N., Pattnaik, S. (2020). Anemia reduction in India: exploring fatigue as a cue to seek treatment. BMJ Global Health. 10:e037471.
20. Sedlander, E., Ladwa, C., Talegawkar, S., Ganjoo, R., Rimal, R. N. (2021). How Gender Norms affect Anemia in Rural, Odisha India: a Qualitative Study. Journal of Nutrition. 111159
21. Sedlander, E. Long, M., Bingenheimer, J., Rimal, R. (2021). Examining intentions to take iron folic acid supplements to inform a behavioral intervention: The Reduction in Anemia through Normative Innovations (RANI) Project. PLOS One. 16(5): e0249646. https://doi.org/10.1371/journal.pone.0249646
22. [dataset] Long, M., Sedlander, E., Bingenheimer, J., Rimal, R. Data from Examining intentions to take iron supplements to inform a behavioral intervention: The Reduction in Anemia through Normative Innovations (RANI) Project, 2019. https://scholarspace.library.gwu.edu/work/gx41mj67p
23. Yilma, H., Sedlander, E., Rimal, R. N., Pant, I. Munjral, A., Mohanty, S. (2020). The Reduction in Anemia through Normative Innovations (RANI) Project: Study Protocol for a Cluster Randomized Controlled Trial in Odisha, India. BMC Public Health, 20(1):203.
24. World Health Organization. (2018). Weekly iron and folic acid supplementation as an anaemia-prevention strategy in women and adolescent girls: lessons learnt from implementation of programmes among non-pregnant women of reproductive age. World Health Organization. https://apps.who.int/iris/handle/10665/274581. Accessed February 4, 2021.
25. Intensified National Iron Plus Initiative (I-NIPI). (2018). Ministry of Health and Family Welfare, Government of India. Anemia Mukt Bharat. https://anemiamuktbharat.info/wp-content/uploads/2019/09/Anemia-Mukt-Bharat-Brochure_English.pdfAccessed February 22, 2021.
26. Sanchis-Gomar F, Cortell-Ballester J, Pareja-Galeano H, Banfi G, Lippi G. Hemoglobin point-of-care testing: the HemoCue system. J Lab Automation. 2012;18(3):198–205.
27. Rogers, W.H. (1993). Regression standard errors in clustered samples. Stata Tech Bull; 13 (Reprinted in Stata Technical Bulletin Reprints, 3: 88–94): 19-23.
28. Viswanath, K., & Kreuter, M. W. (2007). Health disparities, communication inequalities, and eHealth. *American journal of preventive medicine*, *32*(5), S131-S133.
29. Donohue, G. A., Tichenor, P. J., & Olien, C. N. (1975). Mass media and the knowledge gap: A hypothesis reconsidered. *Communication research*, *2*(1), 3-23.
30. Viswanath, K., & Finnegan Jr, J. R. (1996). The knowledge gap hypothesis: Twenty-five years later. *Annals of the International Communication Association*, *19*(1), 187-228.
31. Girma, E., Gebretsadik, L. A., Kaufman, M. R., Rimal, R. N., Morankar, S. N., & Limaye, R. J. (2014). Stigma against people with HIV/AIDS in rural Ethiopia, 2005 to 2011: signs and predictors of improvement. AIDS and Behavior, 18(6), 1046-1053.

For peer review only - http://bmjopen.bmj.com/site/about/guidelines.xhtml

32. Rimal, R. N. (2000). Closing the knowledge-behavior gap in health promotion: The mediating role of self-efficacy. *Health communication*, *12*(3), 219-237.
33. Rimal, R. N., Flora, J. A., & Schooler, C. (1999). Achieving improvements in overall health orientation: Effects of campaign exposure, information seeking, and health media use. Communication Research, 26(3), 322-348.
34. Rimal, R. N., Yilma, H., Sedlander, E., Mohanty, S., Patro, L., Pant, I., Khuntia, S. K., Swain, M., & Behera, S. (2021). Iron folic acid consumption and changing social norms: Cluster randomized field trial, Odisha, India. Bulletin of the World Health Organization, 99(11), 762-772A. doi: 10.2471/BLT.21.285915

For peer review only

Appendix Table 1. Regression coefficients from models predicting midline hemoglobin as a function of treatment assignment, exposure to three intervention components, and control variables

	Model 1	Model 2	Model 3	Model 4	Model 5
RANI Intervention Overall	0.12	0.14	0.11	0.10	0.07
Group education sessions					
1 (vs. 0)		0.11			0.11
2 (vs. 0)		0.19			0.17
3 (vs. 0)		0.14			0.08
4 (vs. 0)		-0.04			-0.11
5 (vs. 0)		-0.03			-0.10
6 (vs. 0)		-0.12			-0.19
7 (vs. 0)		-0.03			-0.03
8 (vs. 0)		0.08			0.06
9 (vs. 0)		-0.08			-0.09
10 (vs. 0)		0.04			0.03
11 (vs. 0)		-0.15			-0.20
Anemia testing					
1 (vs. 0)			0.11		0.12
2 (vs. 0)			-0.13		-0.14
3 (vs. 0)			-0.29		-0.31
4+ (vs. 0)			-0.19		-0.17
RANI Comm videos					
1 (vs. 0)				0.18	0.18
2 (vs. 0)				-0.09	-0.04
3 (vs. 0)				-0.02	0.08
4+ (vs. 0)				0.05	0.20
Control Variables					
Age	-0.00	-0.00	-0.00	-0.00	-0.00
Education	0.02	0.01	0.02*	0.02	0.02
Breastfeeding	-0.16**	-0.16*	-0.16**	-0.16**	-0.16**
Number of children	-0.02	-0.02	-0.02	-0.02	-0.02
Caste/Tribe	-0.39***	-0.39***	-0.39***	-0.39***	-0.39***
Knows anemia status at baseline	-0.00	-0.00	-0.00	-0.00	-0.00
Baseline IFA use	-0.39**	-0.40**	-0.40**	-0.39**	-0.40**
Non-RANI intervention exposure	0.15	0.13	0.15	0.15	0.12
R2	0.03	0.04	0.04	0.04	0.04

For peer review only

CONSORT 2010 checklist of information to include when reporting a randomised trial*

Section/Topic	Item No	Checklist item	Reported on page No
**Title and abstract**			
	1a	Identification as a randomised trial in the title	1
	1b	Structured summary of trial design, methods, results, and conclusions (for specific guidance see CONSORT for abstracts)	1-2
**Introduction**			
Background and objectives	2a	Scientific background and explanation of rationale	2-3
	2b	Specific objectives or hypotheses	3-4
**Methods**			
Trial design	3a	Description of trial design (such as parallel, factorial) including allocation ratio	6-7
	3b	Important changes to methods after trial commencement (such as eligibility criteria), with reasons	n/a
Participants	4a	Eligibility criteria for participants	7-8
	4b	Settings and locations where the data were collected	4
Interventions	5	The interventions for each group with sufficient details to allow replication, including how and when they were actually administered	reported in protocol paper which is cited in text
Outcomes	6a	Completely defined pre-specified primary and secondary outcome measures, including how and when they were assessed	8-9
	6b	Any changes to trial outcomes after the trial commenced, with reasons	n/a
Sample size	7a	How sample size was determined	reported in protocol paper which is cited in text
	7b	When applicable, explanation of any interim analyses and stopping guidelines	n/a
Randomisation:			
Sequence generation	8a	Method used to generate the random allocation sequence	6-7
	8b	Type of randomisation; details of any restriction (such as blocking and block size)	6-7
Allocation	9	Mechanism used to implement the random allocation sequence (such as sequentially numbered containers),	6-7

For peer review only

concealment mechanism		describing any steps taken to conceal the sequence until interventions were assigned	
Implementation	10	Who generated the random allocation sequence, who enrolled participants, and who assigned participants to interventions	reported in protocol paper which is cited in text
Blinding	11a	If done, who was blinded after assignment to interventions (for example, participants, care providers, those assessing outcomes) and how	reported in protocol paper which is cited in text
	11b	If relevant, description of the similarity of interventions	reported in protocol paper which is cited in text
Statistical methods	12a	Statistical methods used to compare groups for primary and secondary outcomes	10-11
	12b	Methods for additional analyses, such as subgroup analyses and adjusted analyses	10-11
**Results**			
Participant flow (a diagram is strongly recommended)	13a	For each group, the numbers of participants who were randomly assigned, received intended treatment, and were analysed for the primary outcome	26
	13b	For each group, losses and exclusions after randomisation, together with reasons	26
Recruitment	14a	Dates defining the periods of recruitment and follow-up	reported in protocol paper which is cited in text
	14b	Why the trial ended or was stopped	n/a
Baseline data	15	A table showing baseline demographic and clinical characteristics for each group	21
Numbers analysed	16	For each group, number of participants (denominator) included in each analysis and whether the analysis was by original assigned groups	21
Outcomes and estimation	17a	For each primary and secondary outcome, results for each group, and the estimated effect size and its precision (such as 95% confidence interval)	11-13; 21-23
	17b	For binary outcomes, presentation of both absolute and relative effect sizes is recommended	11-13; 21-23
Ancillary analyses	18	Results of any other analyses performed, including subgroup analyses and adjusted analyses, distinguishing pre-specified from exploratory	11-13; 21-23

For peer review only

Harms	19	All important harms or unintended effects in each group (for specific guidance see CONSORT for harms)	n/a
**Discussion**			
Limitations	20	Trial limitations, addressing sources of potential bias, imprecision, and, if relevant, multiplicity of analyses	17
Generalisability	21	Generalisability (external validity, applicability) of the trial findings	14-17
Interpretation	22	Interpretation consistent with results, balancing benefits and harms, and considering other relevant evidence	13-17
**Other information**			
Registration	23	Registration number and name of trial registry	2
Protocol	24	Where the full trial protocol can be accessed, if available	6
Funding	25	Sources of funding and other support (such as supply of drugs), role of funders	2

*We strongly recommend reading this statement in conjunction with the CONSORT 2010 Explanation and Elaboration for important clarifications on all the items. If relevant, we also recommend reading CONSORT extensions for cluster randomised trials, non-inferiority and equivalence trials, non-pharmacological treatments, herbal interventions, and pragmatic trials. Additional extensions are forthcoming: for those and for up to date references relevant to this checklist, see www.consort-statement.org.

For peer review only

BMJ Open

How does a social norms-based intervention affect behavior change? Interim Findings from a cluster randomized controlled trial in Odisha, India

Journal:	*BMJ Open*
Manuscript ID	bmjopen-2021-053152.R2
Article Type:	Original research
Date Submitted by the Author:	16-Jun-2022
Complete List of Authors:	Sedlander, Erica; University of California San Francisco<br>Pant, Ichhya; George Washington University School of Public Health and Health Services<br>Bingenheimer, Jeffrey ; George Washington University Department of Health Policy<br>Yilma, Hagere; Boston University<br>Patro, Lipika; IPE Consulting<br>Mohanty, Satya; DCOR Consulting<br>Ganjoo, Rohini; The George Washington University<br>Rimal , Rajiv; Johns Hopkins University Bloomberg School of Public Health
**Primary Subject Heading**:	Global health
Secondary Subject Heading:	Public health, Evidence based practice
Keywords:	PUBLIC HEALTH, NUTRITION & DIETETICS, Public health < INFECTIOUS DISEASES

SCHOLARONE™
Manuscripts

I, the Submitting Author has the right to grant and does grant on behalf of all authors of the Work (as defined in the below author licence), an exclusive licence and/or a non-exclusive licence for contributions from authors who are: i) UK Crown employees; ii) where BMJ has agreed a CC-BY licence shall apply, and/or iii) in accordance with the terms applicable for US Federal Government officers or employees acting as part of their official duties; on a worldwide, perpetual, irrevocable, royalty-free basis to BMJ Publishing Group Ltd ("BMJ") its licensees and where the relevant Journal is co-owned by BMJ to the co-owners of the Journal, to publish the Work in this journal and any other BMJ products and to exploit all rights, as set out in our licence.

The Submitting Author accepts and understands that any supply made under these terms is made by BMJ to the Submitting Author unless you are acting as an employee on behalf of your employer or a postgraduate student of an affiliated institution which is paying any applicable article publishing charge ("APC") for Open Access articles. Where the Submitting Author wishes to make the Work available on an Open Access basis (and intends to pay the relevant APC), the terms of reuse of such Open Access shall be governed by a Creative Commons licence – details of these licences and which Creative Commons licence will apply to this Work are set out in our licence referred to above.

Other than as permitted in any relevant BMJ Author's Self Archiving Policies, I confirm this Work has not been accepted for publication elsewhere, is not being considered for publication elsewhere and does not duplicate material already published. I confirm all authors consent to publication of this Work and authorise the granting of this licence.

For peer review only

Title: How does a social norms-based intervention affect behavior change? Interim findings from a cluster randomized controlled trial in Odisha, India

Authors: Erica Sedlander, DrPH, MPH[1], Ichhya Pant, MPH[2], Jeffrey Bart Bingenheimer, PhD,[2] Hagere Yilma, PhD, MPH[3], Lipika Patro,[4] Satya Mohanty, MS,[5] Rohini Ganjoo, PhD[6], Rajiv N. Rimal, PhD[2, 7]

[1] The University of California San Francisco, Department of Social and Behavioral Sciences, Institute for Health and Aging
[2]The George Washington University, Milken Institute School of Public Health, Department of Prevention and Community Health
[3]Boston University, Sargent College, Department of Health Sciences
[4] IPE Global Limited
[5] DCOR Consulting
[6] The George Washington University, School of Medicine and Health Sciences, Department of Integrated Health Sciences
[7]Johns Hopkins University, Department of Health Behavior and Society

Erica Sedlander: (corresponding author)
University of California San Francisco, Department of Social and Behavioral Sciences, Institute for Health and Aging
esedlander@gwmail.gwu.edu

Erica Sedlander: esedlander@gwmail.gwu.edu
Ichhya Pant: ipant@email.gwu.edu
Jeffrey Bart Bingenheimer: bartbing@gwu.edu
Hagere Yilma hyilma@gwmail.gwu.edu
Lipika Patro: lipika.patro@ipeckd.org
Satya Mohanty: satya.dcor@gmail.com
Rohini Ganjoo: rganjoo@email.gwu.edu
Rajiv N. Rimal: rimal@jhu.edu

For peer review only - http://bmjopen.bmj.com/site/about/guidelines.xhtml

Title: How does a social norms-based intervention affect behavior change? Interim findings from a cluster randomized controlled trial in Odisha, India

Abstract

Background: Behavior change interventions targeting social norms are burgeoning, but researchers have little guidance on what they look like, and which components affect behavior change. The Reduction in Anemia through Normative Innovations (RANI) project designed an intervention to increase iron folic acid consumption in Odisha, India.

Objective: This paper examines the effect of the intervention at midline to understand which components of the RANI intervention affect uptake.

Methods: Using a cluster randomized controlled design, we collected baseline data and midline data six months later from women of reproductive age in the control and treatment arms (n = 3,800) in Angul, Odisha, India. Using nested models, we analyzed data from three different intervention components, monthly community-based testing for anemia, participatory group education sessions, and videos, to determine the extent to which exposure to each of these components accounted for the overall intervention effect on hemoglobin and self-reported IFA use.

Results: Overall, residing in a treatment as opposed to control village had little effect on midline hemoglobin, but increased the odds of taking supplements by 17 times. Exposure to each of the intervention components had a dose-response relationship with self-

reported IFA use. These components, separately and together, accounted for most of the overall effect of treatment assignment on IFA use.

Conclusions: All intervention components increased iron supplement use to differing degrees of magnitude. It appears that a social norms-based approach can result in improving iron folic acid uptake, though improvements in hemoglobin counts were not yet discernible.

Keywords: social norms, behavior change, intervention, anemia

Strengths and limitations of this study

- The intervention is based on formative research, theory, and co-designed with input from community stakeholders
- Data comes from a large, blinded cluster-randomized controlled trial
- Intervention components are qualitatively described and then quantitatively evaluated to decipher their individual effect on iron supplement use
- We rely on self-report for iron supplement use and image recall for participation in intervention activities

Introduction

Social norms, defined as informal rules of behavior considered acceptable in a group or society [1], are increasingly recognized as drivers of or barriers to behavior change. In a review of social norms-based interventions over the last three decades, more than half were published in the last decade [2]. Recent social norms interventions in low to middle income countries have focused on changing harmful behaviors, such as intimate partner violence [3-4], female genital cutting [5], and child marriage [6]. Past research shows that

For peer review only - http://bmjopen.bmj.com/site/about/guidelines.xhtml

people are more likely to engage in a behavior when they believe many others also do so, and when there is a social expectation that they themselves should comply. In the anemia prevention context recent literature indicates a seemingly simple behavior, e.g., taking a weekly iron supplement, is embedded in social and cultural dynamics that dictate which health behaviors are appropriate [7-8].

The theory of normative social behavior (TNSB) highlights the critical role that social norms can play in influencing health behaviors and the circumstances under which they may do so [9-10]. According to the TNSB, the effects on behavior of descriptive norms (i.e., one's perceptions about others' behaviors) and injunctive norms (i.e., one's perceptions of social expectations regarding the behavior) may depend upon other factors at the individual, behavioral, and environmental level [11]. Factors that could either augment or attenuate the effect of social norms messaging on iron folic acid (IFA) use include perceptions that one is at risk of getting anemia, outcome expectations that taking IFA will result in health benefits like preventing anemia or reducing fatigue, and access to and availability of IFA. Therefore, interventions must aim to improve both social norms around a behavior along with potential moderators that may strengthen the relationship between social norms and behaviors. In a 2018 meta-analysis of social norms approaches to behavior change, Dempsey and authors found that the most effective social norms manipulations take place in one's own environment (e.g., a field trial), those that deliver messages in multiple formats, and those that target collectivist groups [12].

Many social norms-based interventions are often complex and resource intensive. To implement them well, program planners need to understand the social context, the social norms around the behavior of interest, and barriers and facilitators at multiple levels of the socio-ecological model [13,14]. Guided by the TNSB, the Reduction in Anemia through Normative Innovations (RANI) Project conducted formative research to delineate significant facilitators to and barriers of iron folic acid consumption in Odisha, India [8].

For peer review only - http://bmjopen.bmj.com/site/about/guidelines.xhtml

Findings from the formative work led to the design of the intervention, which included three components: (a) hands-on participatory learning modules conducted in small groups, (b) dissemination of short videos focusing on iron consumption norms, and (c) monthly hemoglobin testing for anemia, followed by public display of (anonymized) community results.

In this paper, we seek to determine which intervention components impact iron folic acid consumption six months later at midline. This knowledge allowed us to adapt the intervention implementation according to empirical findings. Since social and political realities on the ground cannot be fully predicted at the outset of any given field trial [15], we deemed this approach more preferable than simply implementing a static intervention based on *a priori* data. Thus, the primary goal of this paper is to examine the extent to which each intervention component contributed to the overall effects of the intervention. In addition, because the influence of the intervention may be different across subgroups, we also examine how susceptibility to intervention impact varied by age, caste, education, and communication activity. We hope that delineating the effects of each intervention component will provide guidance for future social norms-based intervention designs. As Davis et al. ([69], p. 2218) argue, "while the social norms approach is based in a rich theory, the theory does little to illuminate implementation details of interventions [16]."

Methods

This study was approved by the George Washington University Institutional Review Board (FWA00005945), Sigma Science and Research, an independent IRB located in New Delhi, India, and the Indian Council for Medical Research's Health Ministry's Screening Committee.

Patient and Public Involvement Statement

Key stakeholders from the community where the intervention was implemented participated in a two-day convening in Bhubaneswar, Odisha to co-design the intervention. Community health workers helped to implement the intervention and disseminate findings back to the community. Patients were not involved in the study – participants were living in the villages where the intervention took place.

Study Setting

Nestled in the eastern coast of India, the state of Odisha is predominantly rural. Most residents (95%) are Hindu, with 23% belonging to specific tribes and practicing tribal culture (NFHS 2015-2016). At approximately 2.1 children per woman, the total fertility rate sits fairly low. Our focal district, Angul, one of 30 in the state of Odisha, has almost 2,000 villages with a total population of just over 1.2 million (Government of Odisha, 2019). Men's literacy rate (87%) is higher than that of women's (70%). Almost a quarter of girls (22%) marry before age 18 and around half of married women of reproductive age use modern methods of family planning [17].

Intervention Development

To develop the RANI intervention, we conducted formative research that examined social norms around IFA use. Between March and May 2018, we collected data from four villages in the two adjacent blocks (administrative units below the district) where the

For peer review only - http://bmjopen.bmj.com/site/about/guidelines.xhtml

intervention took place (Kishorenagar and Athamalik). We conducted 16 focus groups and 21 individual interviews (n = 148), stratified by age and gender, with women of reproductive age, husbands, mothers-in-law, and key informants. To explore women's social norms within the focus groups, we used vignettes, short stories about theoretical characters that also live in a rural village in Angul, India [18]. Vignettes can also help uncover if social sanctions exist and unpack existing social norms. Four researchers, two from India and two from the United States, analyzed transcripts using NVivo v.12 to identify barriers and facilitators to IFA use.

We found that social norms and available services varied substantially for pregnant women, non-pregnant women, and adolescents. Specifically, we found that most participants believed only pregnant women and adolescents in school consume IFA (descriptive norms). Participants also stated that only pregnant women and those diagnosed with anemia should be taking IFA regularly (injunctive norms) and we found that frontline health workers only distributed IFA to pregnant women. Adolescents enrolled in school can also obtain them weekly [8].

Non-pregnant women (our sample for this paper) were not receiving IFA from frontline health workers. Indeed, barriers faced by non-pregnant women were significant: they needed to visit a health center, get tested for anemia, and then obtain the IFA if they were diagnosed as anemic. We also found that risk perception was low, with most participants believing that only "a handful of women" in their community had anemia when, in reality, more than half of women are anemic. When anemia was referenced, we found that participants were primarily referring to severe anemia, not its mild or moderate forms [8].

Our findings also revealed that inequitable gender norms were an upstream barrier to women's accessing and adhering to IFA supplements. Specifically, women prioritized their family's health and well-being over their own, normalized fatigue as part of a

For peer review only - http://bmjopen.bmj.com/site/about/guidelines.xhtml

woman's plight, and given that they often do all of the household work while also working outside of the home, they lacked time (and often autonomy) to visit a health center on their own [19-20].

We used findings from the qualitative research, past literature on anemia reduction efforts, and the TNSB to design the RANI intervention. A three-day convening in Bhubaneswar, Odisha, was held where we invited frontline health workers from the community, anemia researchers, and program planners to co-design an effective social norms-based intervention. Finally, we used quantitative data collected at baseline to refine the intervention design. Specifically, we validated qualitative findings that very few non-pregnant women were taking IFA with a specific percentage (less than 3% of women) despite guidelines that all women should take them regularly to prevent anemia [21]. Therefore, we decided to focus more on injunctive norms messaging (that all women of reproductive age should take IFA) rather than descriptive norms messaging (that women are taking IFA) in the beginning of the intervention.

The RANI Project intervention comprises three main components, each tapping into social norms differently: participatory learning modules; RANI Comms (videos), and community hemoglobin testing. A full description of all RANI intervention activities including specifics about each component can be found in **Table 1**. All RANI project data is stored in an online data repository [22].

Study Design

Data for this study report interim midterm findings from the main trial of the RANI project [23]. The RANI project uses a cluster randomized controlled trial to evaluate the efficacy of a norms-based intervention to increase IFA use and reduce anemia in Odisha, India. The RANI project selected two blocks within the Angul district in the state of Odisha. We grouped contiguous villages

For peer review only - http://bmjopen.bmj.com/site/about/guidelines.xhtml

in these blocks into clusters, which we then randomly assigned to either the treatment or control arm. Villages in the treatment arm were exposed to the RANI intervention, while villages in the control arm continued with “care-as-usual”. We created clusters to minimize contamination; clusters were separated by either a natural buffer (i.e., mountain or river) or a village that was neither in the treatment nor in the control arm. This process resulted in a total of 89 clusters from 239 villages. We then segmented clusters by the proportion of caste/ethnic groups (in India they are called scheduled castes and scheduled tribes) and then selected 3 per stratum, for a total of 15 clusters per arm to be included in data analysis (which comprised 81 villages).

In this paper, we report results from the baseline and midterm assessment, which is a longitudinal study from both the treatment and control clusters. The response rate for the midline questionnaire was 96.2%. Interviewers visited homes up to three times and the primary reason for not taking the midline survey was not being home when the interviewers visited their house.

Participants

In each designated village, we first enumerated all households and then randomly selected households for data collection using proportion-to-size principles based on cluster population. From the selected home, one woman of reproductive age (between 15 and 49 years old) was chosen (randomly if more than one woman was eligible in the same home). Although our sample consisted of 3,953 participants, this paper restricts the sample to those who were not pregnant at the time of the midline survey (n = 3,800). We do so because the primary dependent variable, taking iron and folic acid tablets, has been heavily promoted among pregnant women by the Government of India. Pregnant women are also enrolled in the health system, where physicians or community health workers provide free IFA. This is not the case among non-pregnant women, who have not been targeted as IFA recipients on the ground despite WHO

For peer review only - http://bmjopen.bmj.com/site/about/guidelines.xhtml

and Indian government recommendations [24-25]. The demographic profile of participants included in our analysis is shown in Table 2.

Procedure

The baseline data was collected in September 2019 and midline data was collected six months later in February 2020. Local data collectors obtained informed consent from all individual participants included in the study in the local language, Odiya. Participants under the age of 18 were required to obtain the written permission of one parent or legal guardian. Data collectors orally administered a one-on-one survey to all participants, which assessed demographic information, psychosocial factors, and anemia-related behaviors.

Inclusion Criteria

Women were eligible for inclusion in the study if they were between the ages of 15 and 49, spoke Odiya, lived in the data collection villages (either treatment or control), and did not plan to move in the next year (as this is a longitudinal study).

Measures

Dependent Variables. The RANI study was evaluating the efficacy of a norms-based intervention to increase IFA use and reduce anemia. Therefore, our study has two dependent variables: self-reported IFA use and objectively measured serum hemoglobin levels. We measured *IFA use at midline* using the interview question, “Have you ever eaten/taken an iron tablet or syrup.” (The interviewer then held up the packet of IFA tablets for the interviewee to see). We coded this as a dichotomous variable, scored 1 if currently taking and 0 if not or did in the past but stopped now. We obtained *hemoglobin levels* from all participants at midline

For peer review only - http://bmjopen.bmj.com/site/about/guidelines.xhtml

through point-of-care hemoglobin tests using a HemoCue photometer (in line with India's National Family Health Survey methodology). This instrument provides hemoglobin levels immediately and accurately [26].

Independent Variables (Exposure to the Intervention). We examined four independent variables. The first is a dichotomous indicator of *treatment assignment* that takes the value 1 for participants residing in intervention villages and 0 for those residing in control villages. The other three independent variables are participants' self-reports of exposure to different components of the RANI intervention. To measure *exposure to the participatory learning modules*, we took the sum of responses to six questions about how often participants had seen materials from these sessions. We used visual images from the sessions, with higher scores indicating more exposure or more frequent exposure (not seen = 0, seen once or twice = 1, and seen more than twice = 2). Each image came from a different participatory learning module. One question asked whether or not they had participated in any of the games that were also a part of the RANI participatory group sessions (scored as No=0 and Yes=1). The total number corresponds to the number of times participants report having seen a particular image from any of the group sessions, with this being a proxy for the intensity of exposure to group participatory sessions. Less than 1% of participants (*n=28*) marked "don't know" (which was coded as missing). We coded exposure to participatory learning sessions as a continuous variable (range 0-11). We assessed participants' *own anemia testing* (exposure to the anemia testing component of the intervention) with the question, "How many times have you been tested for anemia as part of the RANI intervention in the last six months?" Response options ranged from 0 (never) to 4 (more than 3 times). We also coded testing for anemia as a continuous variable (range 0-4). We measured *exposure to the RANI Comm videos* as the sum of responses to four questions about which of the four videos they had watched. Interviewers shared an image from each video and a

For peer review only - http://bmjopen.bmj.com/site/about/guidelines.xhtml

brief description of the story plot. Responses were treated as a continuous variable and summed across the four videos for a range from 0 to 4.

Control Variables. We asked respondents their *age*, highest completed level of *education*, and whether they belonged to a *scheduled tribe*. *IFA use at baseline* was assessed exactly as described above for midline. We asked respondents about the *number of children* they had and whether or not they were *breastfeeding*. Additionally, to understand if participants had been exposed to another intervention that was not affiliated with RANI (to avoid contamination), we asked participants, "Did you hear anything about nutrition or iron tablets from the Swabhimaan or any other program?" We coded this as a dichotomous variable (No or don't know = 0 and Yes = 1).

Statistical Analysis

We conducted our analyses in three steps. First, we calculated frequencies and descriptive statistics of all key analytic variables by treatment and control arm and obtained p-values testing the null hypothesis of no difference between the two arms via linear and logistic regression for continuous and categorical variables, respectively. These analyses covered several socioeconomic and health-related background variables; amount of exposure to each of the three intervention components between baseline and midline; and hemoglobin levels and self-reported IFA use at midline. The latter provided unadjusted intention-to-treat estimates of the overall RANI treatment effect on these primary endpoints at midline.

Second, we ran linear regressions to examine if exposure to each intervention component varied by select sociodemographic factors: age (32 years or older versus below 32 years old – the halfway point between the age range), belonging to a schedule caste or

For peer review only - http://bmjopen.bmj.com/site/about/guidelines.xhtml

not, and education (completed primary school or not). These analyses were limited to residents of clusters assigned to the RANI intervention arm.

Third, we ran a series of regression models to examine how each intervention component individually and additively affected hemoglobin levels (linear) and IFA use (logistic) at midline and accounted for the overall effect of treatment assignment. For each outcome, Model 1 includes only control variables (age, education, currently breastfeeding, number of children, whether they belong to a scheduled caste or tribe, knowing anemia status at baseline, IFA use at baseline, and whether the participant reported exposure to a non-RANI intervention) and RANI treatment assignment. The coefficients on RANI treatment assignment in these models represent adjusted intention-to-treat estimates for each outcome. In Model 2, we add a set of dummy variables representing levels of exposure to the first RANI intervention component, group education sessions, with zero sessions as the reference category. The dummy variable specification provides flexibility in the nature of the relationship between number of group education sessions attended and the two outcome variables. The coefficients on the dummy variables in this model represent how midline hemoglobin and self-reported IFA use vary in relation to number of group education sessions attended. Moreover, comparison of the coefficient on the RANI treatment assignment variable in this model to the analogous coefficient in Model 1 provides insight into the extent to which group education sessions contributed to the overall effect of RANI. The next two models are similar to Model 2 but using hemoglobin testing (Model 3) and viewing of RANI Comm videos (Model 4) instead of group education session attendance. Finally, Model 5 includes all three intervention components. The coefficients on the intervention component dummy variables in this model provide some insight into the unique contribution of each component, independent of the other two. And comparison of the coefficient on the RANI treatment

For peer review only - http://bmjopen.bmj.com/site/about/guidelines.xhtml

assignment dummy variable in this model to the corresponding coefficient from Model 1 represents the extent to which the three intervention components jointly contributed to the overall RANI intervention effect. We used Stata version 16 to conduct all analyses, with Huber-White clustered standard errors [27] to account for the sampling and cluster randomization design.

Results

Description of the sample included in our study is shown in Panel A of Table 2. Average age was 31 years old and between a quarter and a third of participants were a part of the tribal population. On average, participants completed primary school (6 years of education). Participants in both treatment and control arms had on average more than one child and fewer than two. About 21% of women in both arms were currently breastfeeding. The only statistically significant difference between the treatment and control arms was age. Women in the treatment arm were 31 years old versus 30 years old in the control arm ($p < .05$). All other demographics were not statistically different by treatment and control. Panel B of Table 2 shows that exposure to non-RANI interventions was low in both study arms.

Crude estimates of the effects of the RANI intervention at midline are shown in Panel C of Table 2. Participants from the treatment arm reported more IFA use at midline (32%) compared to the control arm (3%) (p = < .001). However, hemoglobin levels were not statistically different in the two arms (11.7% versus 11.5%) (p =.28) (see Appendix Table 1). Panel D of Table 2 shows that exposure to each of the intervention components was widespread among participants in the treatment arm and low among participants in the control arm. Specifically, 88% of participants in the treatment arm compared to only 12% in the control arm reported that they attended at least one group educational session (p = <.001). Over eighty percent of participants in the treatment arm reported that they

For peer review only - http://bmjopen.bmj.com/site/about/guidelines.xhtml

had been tested at least once as part of the intervention compared to 0% in the control arm (p = <.001). Similarly, 80% of women in the treatment arm reported that they watched one or more RANI Comm videos, compared to less than 1% in the control arm (p = <.001). Lastly, exposure to interventions other than RANI was minimal across both the treatment and control arms (Panel B, 1.2% and 2.1%, respectively).

Table 3 shows that less educated women attended more educational sessions (4.77 compared to 4.46; $p = < .05$) but more educated women had a higher average number of anemia tests(1.32 compared to 1.22; $p = < .05$). Table 3 also shows that neither age nor caste or tribal background was associated with exposure to the intervention.

As there was no crude difference between the treatment and control arms in hemoglobin level at midline (see Panel C of Table 2), we included the analyses linking exposure to specific intervention components to hemoglobin levels as an appendix (See Appendix Table 1). However, nested logistic regression models predicting self-reported IFA use as a function of study arm and exposure to each of the intervention components are presented in Table 4. As seen in Model 1, after controlling for demographic variables and IFA use at baseline, simply being in the treatment arm increased a woman's odds of taking IFA by more than 16 times (OR = 16.94; p = < .001). In Model 2, we added exposure to the group education sessions and found that the odds of taking IFA increased as the number of sessions attended rose. Model 3 suggests that each additional test for anemia also showed an increase in the odds of taking IFA. Model 4 similarly shows that for each RANI Comm video that a woman watched there was an increase in her odds of taking IFA. Model 5 with all intervention components included shows that while all effect sizes drop (including the effect of RANI treatment

For peer review only - http://bmjopen.bmj.com/site/about/guidelines.xhtml

assignment), each component still had a significant effect on IFA use This indicates that each component explains a unique part of the variance in current use of IFA.

Discussion

In this paper we present findings from the midline interim assessment of a multicomponent behavioral intervention intended to increase IFA use and decrease anemia among women of reproductive age in Odisha, India. We find strong evidence that the intervention increased the prevalence of self-reported IFA use among non-pregnant women, but no evidence of an effect of the intervention on average hemoglobin levels in that group. Self-reported exposure to all three intervention components was high among women in the treatment arm, which did not vary substantially by age or membership in a scheduled caste or tribe. However, exposure to anemia testing was higher among more educated women and exposure to group sessions was higher among less educated women. Self-reported exposure to all three intervention components was positively associated with IFA use, and these exposures accounted for most but not all the overall effect of the RANI intervention on IFA use among non-pregnant women.

Given that we saw very little difference in exposure to the intervention components by age, education or caste, it appears that the RANI Project's outreach efforts did not disproportionately favor those with higher privilege in society. Indeed, even though exposure to the intervention did not differ by tribal status or age, in our multivariate models we found some evidence that those with lesser education had higher IFA uptake. We view this finding rather optimistically, given that interventions have the potential for exacerbating existing differences in society along access and socioeconomic lines [28], as has been documented by the literature on the knowledge gap hypothesis [29-30].

For peer review only - http://bmjopen.bmj.com/site/about/guidelines.xhtml

We saw strong evidence that testing women for anemia (through finger-prick hemoglobin counts) was associated with IFA uptake: those who got tested more often were more likely to consume IFA tablets. The reasons for this are not known precisely, but we do have a few explanations. First, it could be that testing revealed to women the low hemoglobin counts, which spurred them to act to improve their scores. Our formative assessment had revealed that feeling weak was often a part of women's self-identity, with women often believing that fatigue was par for the course [19] and that this was part of the gendered norms for women [20].We suspect that, in contrast, an objective measure of iron in the blood, observed through hemoglobin testing, quantified and thus provided precision to a phenomenon woman had come to accept as a vague notion of fatigue. We found that testing was highly demanded and motivational. Indeed, our process evaluation data indicate that, despite a high (60%) coverage rate of anemia testing for women in our intervention catchment areas, we have been unable to meet the demand for testing and repeat testing. If were able to meet the demand, the impact of testing may have been even higher.

A second reason why testing may have motivated women to consume IFA tablets pertains to observational learning around social norms. Due to test results being displayed publicly in the community, and most educational sessions linked the test results with IFA consumption, women likely came to link their consumption behaviors with hemoglobin readings – not only for themselves but also for what they had observed among others in their community, where hemoglobin testing was a monthly event. These events were so popular that, in some villages, the waiting list for testing ran up to 90 names (we were unable to accommodate more than 15 women per village per month, given the study design and resources). This public display of IFA testing and hemoglobin results also normalized women prioritizing their own health potentially shifting gender norms that solely focus on pregnant women's health or the

For peer review only - http://bmjopen.bmj.com/site/about/guidelines.xhtml

health of the family. Although these underlying reasons are somewhat speculative, they do point to the need to study more precisely the link between testing and IFA consumption.

Each educational participatory learning session that women attended significantly increased IFA use. These sessions were designed primarily to raise awareness and improve knowledge about anemia, iron-rich foods, and diet diversity. We also included discussions about gender roles, how eating order in the home disfavors women's health, and the need to remain strong to be able to take care of others. We have few reasons to believe that knowledge in these domains would directly translate into behaviors. However, a significant body of work demonstrates that knowledge about health is a necessary, though not sufficient, condition for behavior change [31 - 32] and that people often hold knowledge in abeyance, to be acted upon at the appropriate time [33]. From this perspective, it seems that educational sessions may have played an important role.

Finally, we found that exposure to the communication videos was also significantly associated with increased IFA uptake. We suspect that the underlying reason for this finding pertains to another normative component of the intervention. The four videos we developed targeted different audiences, including adolescents, husbands, mothers-in-law, and women of reproductive age. As an explicit campaign strategy, each video was shown to each of the parties, including those who were not the explicit target audience for the particular video. This was done so that each group came to understand that the other groups were also being targeted by the intervention. So, for example, adolescent girls saw that there were videos that also addressed men and older women. Similarly, men saw that the videos targeted other men and other women. The overall strategy was to communicate the message that the entire community was engaged in the task of reducing anemia.

For peer review only - http://bmjopen.bmj.com/site/about/guidelines.xhtml

In this paper, we assessed how each of the intervention components affected IFA use. This helps elucidate where this intervention should focus for the remaining trial, provides a clear picture of dose response, and highlights where other social norms program implementors may want to focus their efforts. In another midline paper, we assessed the effect of social norms on IFA use and found that changes in descriptive and collective norms (but not injunctive norms) were associated with changes in self-reported IFA use [34].

It is also important to discuss the lack of an effect of the RANI intervention on hemoglobin use despite an increase in self-reported IFA use. One plausible explanation of these disparate findings is that the increase in IFA use among members of the treatment arm is real but is not sufficient in magnitude or duration to produce a corresponding increase in hemoglobin levels. Alternatively, it could be attributable to differential misreporting of IFA use among treatment group members, a form of courtesy or social desirability bias. Findings from the end line data collection, planned to take place at the end of the project, may help to adjudicate between these two explanations.

Of course, our study is not without limitations. First and foremost, while treatment assignment is objectively measured on the basis of project administrative data, exposure to the three intervention components is self-reported and may be subject to some level of misreporting. Furthermore, it is possible that women who participate in the intervention may be more motivated to change behavior in general and are already more inclined to take IFA. Therefore, Table 4 could overstate the effects on IFA use of exposure to the intervention components, as well as the contributions of those components to the overall RANI effect. However, IFA use is still a result of participating in the RANI intervention, as participation led to IFA use so we may have simply captured women who were

For peer review only - http://bmjopen.bmj.com/site/about/guidelines.xhtml

farther along in their readiness to change. Additionally, although our study used a representative sample of areas and women in our focal areas, findings may not be generalizable outside of that setting.

Despite these limitations, our study has strengths: the intervention is based on formative research; it is co-designed by stakeholders from the area; we use a theoretically driven and adaptive approach; and the study design itself is robust, with an underlying randomization to experimental arms.

Conclusion

Our findings show that a social norms-based intervention can be successful in increasing IFA use. They also demonstrate that unique intervention components separately and altogether impact this success. All three components appear to contribute something to the overall effect of the intervention. While all three intervention components tapped into social norms messaging, hemoglobin testing provided individual health information, village level health information, a comparison to other villages, and changes in health information over time. This multi-level component, coupled with the other two components, may help women reach the tipping point to take and adhere to IFA. While IFA use shows promise, hemoglobin levels may need more time to show significant changes, especially among non-pregnant women who were not taking IFA and who only take it weekly. End line results will elucidate more information about the full RANI intervention effects.

For peer review only - http://bmjopen.bmj.com/site/about/guidelines.xhtml

Trial Registration
This trial was registered with Clinical Trial Registry- India (CTRI) (CTRI/2018/10/016186) on 29 October 2018.

Funding
This work was supported by a grant from The Bill and Melinda Gates Foundation (OPP1182519) to the George Washington University, Rajiv N. Rimal, principal investigator. The funders had no role in study design, data collection and analysis, decision to publish, or preparation of the manuscript.

Author Contribution Statement
ES: Conceptualization, methodology, analysis, writing – original draft
IP: Conceptualization, methodology, writing – review & editing
JB: Methodology, writing – review & editing
HY: Methodology, data curation, writing – review & editing
LP: Project administration, supervision, writing – review & editing
SM: Project administration, supervision, writing – review & editing
RG: writing – review & editing
RR: Conceptualization, methodology, supervision, funding acquisition, writing – review & editing

The authors have no competing interests.

Data sharing statement:

Data used for this study can be found in our online data repository: GW Scholarspace (https://scholarspace.library.gwu.edu/) and can also be requested from the first author.

For peer review only - http://bmjopen.bmj.com/site/about/guidelines.xhtml

Table 1. Description of RANI intervention activities

**Group participatory learning sessions**<br>We developed ten one-hour-long group participatory learning modules on various topics related to anemia prevention, including iron folic acid supplementation, diet diversity, and social norms/gender norms that may impact a woman's ability to take iron supplements. These monthly group participatory learning sessions are delivered through in-person activities and games. Women and their social networks (e.g., husbands and mothers-in-law) were all invited to participate so that women and those important to her are being exposed to the same messaging. We covered four participatory learning modules before midline data collection.
**RANI Comm videos**<br>We created four RANI Comm videos in the local language, Odiya, with local residents as actors. These videos highlighted the stories of women overcoming barriers related to IFA consumption and other social norms prevalent in the area. The videos were shown on smartphones and tablets to both individuals and small groups. The videos targeted various audiences (pregnant women, non-pregnant women, husbands, and mothers-in-law) and addressed barriers and facilitators to IFA use that we identified in the formative research

For peer review only - http://bmjopen.bmj.com/site/about/guidelines.xhtml

followed by group discussion sessions. Descriptive and injunctive norms messages around IFA consumption were included in each storyline. An example of injunctive norms messaging in the storyline is a mother-in-law expressing that her daughter-in-law should be taking care of herself too, not just looking after the family, and should be taking weekly IFA to avoid anemia even if she is not pregnant. A new video was rolled out every 2-3 months. The viewing time for one video was approximately 15 minutes. Even though videos were 3-4 minutes long, the pre and post viewing discussion took an additional 10 minutes. Participants could have seen the same video more than once.
**Community based hemoglobin testing**   We also conducted monthly anemia testing of the women using a digital Hemocue meter. These instant results are shared at the individual, group, and village levels with the help of blood shaped cards (different colors indicating anemia severity) and infographics appropriate for a low literacy population. Monthly community-based testing was followed by a discussion about trends in anemia and village-level comparisons (based on the hemoglobin readings) with neighboring communities at both the individual and community levels. This provides ipsative feedback (information people receive about their ongoing progress over time), normative

For peer review only

feedback (information about the particular individual's achievements relative to those of her social peer), and aspirational feedback (comparisons people make between their current state of affairs and the goals they may have set for themselves). Testing sessions lasted for an hour as community/group testing was followed up by demonstration of results and behavioral nudges for improving their Hb count.

* Community Facilitators of the RANI team delivered the intervention. However, IFA tablets were provided by the community health workers. RANI started off using key influencers in the village like front line health workers to gather people for the intervention. However, as our facilitators became familiar with the community, we also accepted RANI volunteers (women from the community) who facilitated this. Some of the interventions were delivered at the household level also.

For peer review only

Table 2. Description of Participants by Study Arm

	Treatment (n=1874)	Control (n=1867)	p-value
Demographic			
Age (years)	31.2	30.5	0.022
Part of tribal pop	23.6%	31.7%	0.267
Education (years)	6.07	6.17	0.749
Breastfeeding at baseline	21.4%	21.7%	0.836
Number of children at baseline	1.69	1.68	0.851
Exposure to non-RANI interventions	1.2%	2.1%	0.283
Dependent variables			
IFA use at midline	32.0%	3.0%	<0.001
Hemoglobin at midline (g/dl)	11.66	11.52	0.281
Exposure to RANI intervention components			
Number of group education sessions attended	4.60	0.23	<0.001
Attended at least one group education session	88.6%	12.1%	<0.001
Number of hemoglobin tests undergone	1.28	0.00	<0.001
Tested for hemoglobin at least once	81.4%	0.0%	<0.001
Number of RANI Comm videos views	2.46	0.00	<0.001
Viewed at least one RANI Comm video	79.8%	0.2%	<0.001

Notes: We ran regression analyses to test the statistical significance between treatment versus control arms on demographic variables. Significant cells are in bold. The other non-RANI related intervention includes anemia related education as one component that may have had some overlap with our intervention. We included this as a control in our models to ensure that any changes in behavior are a result of our intervention alone.

For peer review only - http://bmjopen.bmj.com/site/about/guidelines.xhtml

Table 3. Exposure to intervention components by age, education, and caste (treatment arm participants)

	Average number of group educational sessions attended	Average number of hemoglobin tests undergone	Average number pf RANI Comm videos seen
Age			
Less than 32 years	4.48 (SD: 2.82)	1.33 (SD: 1.04)	2.41 (SD: 1.55)
32 years or more	4.74 (SD: 2.95)	1.22 (SD: 0.95)	2.53 (SD: 1.55)
p-value	0.219	0.056	0.119
Education			
Up to completed primary	4.77 (SD: 2.88)	1.22 (0.93)	2.55 (SD: 1.55)
More than completed prim	4.46 (SD: 2.88)	1.32 (1.05)	2.40 (SD: 1.55)
p-value	0.040	0.033	0.132
Scheduled Tribe or Caste			
Yes	4.54 (SD: 2.87)	1.28 (SD: 1.00)	2.44 (SD: 1.56)
No	4.78 (SD: 2.90)	1.29 (SD: 1.01)	2.55 (SD: 1.52)
p-value	0.429	0.899	0.407

*SD = Standard deviations

For peer review only

Table 4. Adjusted odds ratios from logistic regression models predicting self-reported IFA use at midline as a function of treatment assignment, exposure to three intervention components, and control variables

	Model 1	Model 2	Model 3	Model 4	Model 5
RANI Intervention Overall	16.94***	5.24***	3.62***	5.16***	2.25*
Group education sessions					
1 (vs. 0)		1.50			1.17
2 (vs. 0)		2.89***			1.71*
3 (vs. 0)		2.94***			1.37
4 (vs. 0)		3.05***			1.30
5 (vs. 0)		3.80***			1.43
6 (vs. 0)		5.37***			1.76*
7 (vs. 0)		5.39***			1.39
8 (vs. 0)		8.53***			2.41*
9 (vs. 0)		8.16***			2.30**
10 (vs. 0)		9.23***			2.23*
11 (vs. 0)		11.97***			2.81**
Anemia testing					
1 (vs. 0)			4.91***		3.07***
2 (vs. 0)			6.93***		3.72***
3 (vs. 0)			11.94***		5.65***
4 (vs. 0)			12.91***		4.90***
RANI Comm videos					
1 (vs. 0)				2.30***	1.44
2 (vs. 0)				2.85***	1.59
3 (vs. 0)				3.57***	1.72*
4 (vs. 0)				5.66***	2.39***
Control Variables					
Age	0.99	0.99	1.00	0.99	0.99
Education	0.98	0.97	0.97	0.98	0.97
Breastfeeding	1.35*	1.37*	1.41*	1.41**	1.43**
Number of children	0.99	0.96	0.96	0.97	0.95
Caste/Tribe	1.31*	1.26	1.31*	1.28	1.26

For peer review only - http://bmjopen.bmj.com/site/about/guidelines.xhtml

Knows anemia status at baseline	1.00	1.00	1.00	1.00	1.00
Baseline IFA use	2.59***	2.94***	3.30***	2.87***	3.36***
Non-RANI intervention exposure	1.85	1.28	1.57	1.59	1.36
Pseudo R2	0.20	0.24	0.24	0.23	0.26

Notes: *$p < .05$, **$p < .01$, ***$p < .00$

Ethical Approval Statement

This study was approved by the George Washington University Institutional Review Board (FWA00005945), Sigma Science and Research, an independent IRB located in New Delhi, India, and the Indian Council for Medical Research's Health Ministry's Screening Committee.

For peer review only - http://bmjopen.bmj.com/site/about/guidelines.xhtml

References

1. Reno, R. R., Cialdini, R. B., & Kallgren, C. A. (1993). The trans situational influence of social norms. *Journal of personality and social psychology*, *64*(1), 104.
2. Yemin, P., Fei, M., Lahlou, S., & Levy, S. (2019). Using social norms to change behavior and increase sustainability in the real world: A systematic review of the literature. Sustainability, 11(20), 5847.
3. Simone Francis, Prabin Nanicha Shrestha, Binita Shrestha, Gemma Ferguson , Brian Batayeh, Monique Hennink & Cari Jo Clark (2020): The Influence of Organised Diffusion on Social Norms Change: Addressing Intimate Partner Violence in Nepal, Global Public Health, DOI: 10.1080/17441692.2020.1845767
4. Clark, C. J., Bergenfeld, I., Batayeh, B., Ferguson, G., Shrestha, B., & Shrestha, P. N. (2019). Intimate partner violence in Nepal: Change starts at home study, what works to prevent violence: A global programme to prevent violence against women and girls evidence brief. South Africa Medical Research Council.
5. Shell-Duncan, B., Wander, K., Hernlund, Y., & Moreau, A. (2011). Dynamics of change in the practice of female genital cutting in Senegambia: testing predictions of social convention theory. Social science & medicine (1982), 73(8), 1275–1283. https://doi.org/10.1016/j.socscimed.2011.07.022
6. Vaitla, Bapu, Alice Taylor, Julia Van Horn, and Ben Cislaghi. 2017. Social Norms and Girls' Well-Being: Linking Theory and Practice. Washington, D.C.: Data2X.
7. Nagata, J. M., Gatti, L. R., & Barg, F. K. (2012). Social determinants of iron supplementation among women of reproductive age: a systematic review of qualitative data. Maternal & child nutrition, 8(1), 1-18.
8. Sedlander, E., Long, M., Mohanty, S., Munjral, A., Bingenheimer, J., Yilma, H., Rimal, R. N. (2020). Moving Beyond Individual Barriers and Identifying Multi-level Strategies to Address Anemia in Rural India. BMC Public Health, 20:457.
9. Rimal, R. N., Lapinski, M. K., Cook, R. J., & Real, K. (2005). Moving toward a theory of normative influences: How perceived benefits and similarity moderate the impact of descriptive norms on behaviors. Journal of health communication, 10(5), 433-450.
10. Rimal, R. N., & Real, K. (2005). How behaviors are influenced by perceived norms: A test of the theory of normative social behavior. Communication research, 32(3), 389-414.
11. Chung, A., and Rimal, R. N. (2016). Social norms: a review. *Rev. Commun. Res.* 4, 1–29. doi: 10.12840/issn.2255-4165.2016.04.01.008
12. Dempsey, R., McAlaney, J., Bewick, B. (2018). A Critical Appraisal of the Social Norms Approach as an Interventional Strategy for Health-Related Behavior and Attitude Change. Frontiers in Psychology, 6. https://doi.org/10.3389/fpsyg.2018.02180
13. Cislaghi, B., Nkwi, P., Mackie, G & Shakya, H. (2020). Why context matters for social norms interventions: The case of child marriage in Cameroon, Global Public Health, 15:4, 532-543, DOI: 10.1080/17441692.2019.1704818
14. Edberg, M., & Krieger, L. (2020). Recontextualizing the social norms construct as applied to health promotion.. *SSM-Population Health, 10* (). http://dx.doi.org/10.1016/j.ssmph.2020.100560
15. Pant, I., Patro, L., Sedlander, E., Gupta, K., Chandarana, S., Rimal, R. Monitor to Innovate with Feedback Loops: Process Evaluation Protocol for an Anemia Prevention Intervention. Study Protocol. Research Square. 10.21203/rs.3.rs-389608/v1

For peer review only - http://bmjopen.bmj.com/site/about/guidelines.xhtml

16. Davis, C.A.; Heiman, J.R.; Menczer, F. A Role for Network Science in Social Norms Intervention. Procedia Comput. Sci. 2015, 51, 2217–2226.
17. International Institute for Population Sciences (IIPS) and ICF. National family health survey (NFHS-4), India, 2015–16: Odisha. Mumbai: IIPS; 2017
18. Gourlay, A., G. Mshana, I. Birdthistle, G. Bulugu, B. Zaba, and M. Urassa. 2014. "Using vignettes in qualitative research to explore barriers and facilitating factors to the uptake of prevention of mother-to-child transmission services in rural Tanzania: A critical analysis," BMC Medical Research Methodology 14(1): 21.
19. Yilma, H., Sedlander, E., Rimal, R. N., Pattnaik, S. (2020). Anemia reduction in India: exploring fatigue as a cue to seek treatment. BMJ Global Health. 10:e037471.
20. Sedlander, E., Ladwa, C., Talegawkar, S., Ganjoo, R., Rimal, R. N. (2021). How Gender Norms affect Anemia in Rural, Odisha India: a Qualitative Study. Journal of Nutrition. 111159
21. Sedlander, E. Long, M., Bingenheimer, J., Rimal, R. (2021). Examining intentions to take iron folic acid supplements to inform a behavioral intervention: The Reduction in Anemia through Normative Innovations (RANI) Project. PLOS One. 16(5): e0249646. https://doi.org/10.1371/journal.pone.0249646
22. [dataset] Long, M., Sedlander, E., Bingenheimer, J., Rimal, R. Data from Examining intentions to take iron supplements to inform a behavioral intervention: The Reduction in Anemia through Normative Innovations (RANI) Project, 2019. https://scholarspace.library.gwu.edu/work/gx41mj67p
23. Yilma, H., Sedlander, E., Rimal, R. N., Pant, I. Munjral, A., Mohanty, S. (2020). The Reduction in Anemia through Normative Innovations (RANI) Project: Study Protocol for a Cluster Randomized Controlled Trial in Odisha, India. BMC Public Health, 20(1):203.
24. World Health Organization. (2018). Weekly iron and folic acid supplementation as an anaemia-prevention strategy in women and adolescent girls: lessons learnt from implementation of programmes among non-pregnant women of reproductive age. World Health Organization. https://apps.who.int/iris/handle/10665/274581. Accessed February 4, 2021.
25. Intensified National Iron Plus Initiative (I-NIPI). (2018). Ministry of Health and Family Welfare, Government of India. Anemia Mukt Bharat. https://anemiamuktbharat.info/wp-content/uploads/2019/09/Anemia-Mukt-Bharat-Brochure_English.pdfAccessed February 22, 2021.
26. Sanchis-Gomar F, Cortell-Ballester J, Pareja-Galeano H, Banfi G, Lippi G. Hemoglobin point-of-care testing: the HemoCue system. J Lab Automation. 2012;18(3):198–205.
27. Rogers, W.H. (1993). Regression standard errors in clustered samples. Stata Tech Bull; 13 (Reprinted in Stata Technical Bulletin Reprints, 3: 88–94): 19-23.
28. Viswanath, K., & Kreuter, M. W. (2007). Health disparities, communication inequalities, and eHealth. *American journal of preventive medicine*, *32*(5), S131-S133.
29. Donohue, G. A., Tichenor, P. J., & Olien, C. N. (1975). Mass media and the knowledge gap: A hypothesis reconsidered. *Communication research*, *2*(1), 3-23.
30. Viswanath, K., & Finnegan Jr, J. R. (1996). The knowledge gap hypothesis: Twenty-five years later. *Annals of the International Communication Association*, *19*(1), 187-228.
31. Girma, E., Gebretsadik, L. A., Kaufman, M. R., Rimal, R. N., Morankar, S. N., & Limaye, R. J. (2014). Stigma against people with HIV/AIDS in rural Ethiopia, 2005 to 2011: signs and predictors of improvement. AIDS and Behavior, 18(6), 1046-1053.

For peer review only - http://bmjopen.bmj.com/site/about/guidelines.xhtml

32. Rimal, R. N. (2000). Closing the knowledge-behavior gap in health promotion: The mediating role of self-efficacy. *Health communication*, *12*(3), 219-237.
33. Rimal, R. N., Flora, J. A., & Schooler, C. (1999). Achieving improvements in overall health orientation: Effects of campaign exposure, information seeking, and health media use. Communication Research, 26(3), 322-348.
34. Rimal, R. N., Yilma, H., Sedlander, E., Mohanty, S., Patro, L., Pant, I., Khuntia, S. K., Swain, M., & Behera, S. (2021). Iron folic acid consumption and changing social norms: Cluster randomized field trial, Odisha, India. Bulletin of the World Health Organization, 99(11), 762-772A. doi: 10.2471/BLT.21.285915

For peer review only

Appendix Table 1. Regression coefficients from models predicting midline hemoglobin as a function of treatment assignment, exposure to three intervention components, and control variables

	Model 1	Model 2	Model 3	Model 4	Model 5
RANI Intervention Overall	0.12	0.14	0.11	0.10	0.07
Group education sessions					
1 (vs. 0)		0.11			0.11
2 (vs. 0)		0.19			0.17
3 (vs. 0)		0.14			0.08
4 (vs. 0)		-0.04			-0.11
5 (vs. 0)		-0.03			-0.10
6 (vs. 0)		-0.12			-0.19
7 (vs. 0)		-0.03			-0.03
8 (vs. 0)		0.08			0.06
9 (vs. 0)		-0.08			-0.09
10 (vs. 0)		0.04			0.03
11 (vs. 0)		-0.15			-0.20
Anemia testing					
1 (vs. 0)			0.11		0.12
2 (vs. 0)			-0.13		-0.14
3 (vs. 0)			-0.29		-0.31
4+ (vs. 0)			-0.19		-0.17
RANI Comm videos					
1 (vs. 0)				0.18	0.18
2 (vs. 0)				-0.09	-0.04
3 (vs. 0)				-0.02	0.08
4+ (vs. 0)				0.05	0.20
Control Variables					
Age	-0.00	-0.00	-0.00	-0.00	-0.00
Education	0.02	0.01	0.02*	0.02	0.02
Breastfeeding	-0.16**	-0.16*	-0.16**	-0.16**	-0.16**
Number of children	-0.02	-0.02	-0.02	-0.02	-0.02
Caste/Tribe	-0.39***	-0.39***	-0.39***	-0.39***	-0.39***
Knows anemia status at baseline	-0.00	-0.00	-0.00	-0.00	-0.00
Baseline IFA use	-0.39**	-0.40**	-0.40**	-0.39**	-0.40**
Non-RANI intervention exposure	0.15	0.13	0.15	0.15	0.12
R2	0.03	0.04	0.04	0.04	0.04

For peer review only

CONSORT 2010 checklist of information to include when reporting a randomised trial*

Section/Topic	Item No	Checklist item	Reported on page No
**Title and abstract**			
	1a	Identification as a randomised trial in the title	1
	1b	Structured summary of trial design, methods, results, and conclusions (for specific guidance see CONSORT for abstracts)	1-2
**Introduction**			
Background and objectives	2a	Scientific background and explanation of rationale	2-3
	2b	Specific objectives or hypotheses	3-4
**Methods**			
Trial design	3a	Description of trial design (such as parallel, factorial) including allocation ratio	6-7
	3b	Important changes to methods after trial commencement (such as eligibility criteria), with reasons	n/a
Participants	4a	Eligibility criteria for participants	7-8
	4b	Settings and locations where the data were collected	4
Interventions	5	The interventions for each group with sufficient details to allow replication, including how and when they were actually administered	reported in protocol paper which is cited in text
Outcomes	6a	Completely defined pre-specified primary and secondary outcome measures, including how and when they were assessed	8-9
	6b	Any changes to trial outcomes after the trial commenced, with reasons	n/a
Sample size	7a	How sample size was determined	reported in protocol paper which is cited in text
	7b	When applicable, explanation of any interim analyses and stopping guidelines	n/a
Randomisation:			
Sequence generation	8a	Method used to generate the random allocation sequence	6-7
	8b	Type of randomisation; details of any restriction (such as blocking and block size)	6-7
Allocation	9	Mechanism used to implement the random allocation sequence (such as sequentially numbered containers),	6-7

For peer review only - http://bmjopen.bmj.com/site/about/guidelines.xhtml

concealment mechanism		describing any steps taken to conceal the sequence until interventions were assigned	
Implementation	10	Who generated the random allocation sequence, who enrolled participants, and who assigned participants to interventions	reported in protocol paper which is cited in text
Blinding	11a	If done, who was blinded after assignment to interventions (for example, participants, care providers, those assessing outcomes) and how	reported in protocol paper which is cited in text
	11b	If relevant, description of the similarity of interventions	reported in protocol paper which is cited in text
Statistical methods	12a	Statistical methods used to compare groups for primary and secondary outcomes	10-11
	12b	Methods for additional analyses, such as subgroup analyses and adjusted analyses	10-11
**Results**			
Participant flow (a diagram is strongly recommended)	13a	For each group, the numbers of participants who were randomly assigned, received intended treatment, and were analysed for the primary outcome	26
	13b	For each group, losses and exclusions after randomisation, together with reasons	26
Recruitment	14a	Dates defining the periods of recruitment and follow-up	reported in protocol paper which is cited in text
	14b	Why the trial ended or was stopped	n/a
Baseline data	15	A table showing baseline demographic and clinical characteristics for each group	21
Numbers analysed	16	For each group, number of participants (denominator) included in each analysis and whether the analysis was by original assigned groups	21
Outcomes and estimation	17a	For each primary and secondary outcome, results for each group, and the estimated effect size and its precision (such as 95% confidence interval)	11-13; 21-23
	17b	For binary outcomes, presentation of both absolute and relative effect sizes is recommended	11-13; 21-23
Ancillary analyses	18	Results of any other analyses performed, including subgroup analyses and adjusted analyses, distinguishing pre-specified from exploratory	11-13; 21-23

For peer review only

For peer review only - http://bmjopen.bmj.com/site/about/guidelines.xhtml

Harms	19	All important harms or unintended effects in each group (for specific guidance see CONSORT for harms)	n/a
**Discussion**			
Limitations	20	Trial limitations, addressing sources of potential bias, imprecision, and, if relevant, multiplicity of analyses	17
Generalisability	21	Generalisability (external validity, applicability) of the trial findings	14-17
Interpretation	22	Interpretation consistent with results, balancing benefits and harms, and considering other relevant evidence	13-17
**Other information**			
Registration	23	Registration number and name of trial registry	2
Protocol	24	Where the full trial protocol can be accessed, if available	6
Funding	25	Sources of funding and other support (such as supply of drugs), role of funders	2

*We strongly recommend reading this statement in conjunction with the CONSORT 2010 Explanation and Elaboration for important clarifications on all the items. If relevant, we also recommend reading CONSORT extensions for cluster randomised trials, non-inferiority and equivalence trials, non-pharmacological treatments, herbal interventions, and pragmatic trials. Additional extensions are forthcoming: for those and for up to date references relevant to this checklist, see www.consort-statement.org.

For peer review only
